# The oak gene expression atlas: insights into Fagaceae genome evolution and the discovery of genes regulated during bud dormancy release

**DOI:** 10.1186/s12864-015-1331-9

**Published:** 2015-02-21

**Authors:** Isabelle Lesur, Grégoire Le Provost, Pascal Bento, Corinne Da Silva, Jean-Charles Leplé, Florent Murat, Saneyoshi Ueno, Jerôme Bartholomé, Céline Lalanne, François Ehrenmann, Céline Noirot, Christian Burban, Valérie Léger, Joelle Amselem, Caroline Belser, Hadi Quesneville, Michael Stierschneider, Silvia Fluch, Lasse Feldhahn, Mika Tarkka, Sylvie Herrmann, François Buscot, Christophe Klopp, Antoine Kremer, Jérôme Salse, Jean-Marc Aury, Christophe Plomion

**Affiliations:** INRA, UMR1202, BIOGECO, F-33610, Cestas, France; HelixVenture, F-33700, Mérignac, France; CEA-Institut de Génomique, GENOSCOPE, Centre National de Séquençage, 2 rue Gaston Crémieux, CP5706, F-91057, Evry Cedex, France; Forestry and Forest Products Research Institute, Department of Forest Genetics, Tree Genetics Laboratory, 1 Matsunosato, Tsukuba, Ibaraki 305-8687 Japan; University Bordeaux, BIOGECO, UMR1202, F-33170, Talence, France; INRA, UR0588 Amélioration Génétique et Physiologie Forestières, F-45075, Orléans, France; INRA/UBP UMR 1095, Laboratoire Génétique, Diversité et Ecophysiologie des Céréales, F-63039, Clermont-Ferrand, France; CIRAD, UMR AGAP, F-34398, Montpellier, France; Plateforme bioinformatique Toulouse Midi-Pyrénées, UBIA, INRA, F-31326, Auzeville Castanet-Tolosan, France; INRA, Unité de Recherche Génomique Info (URGI), F78026, Versailles, France; AIT Austrian Institute of Technology GmbH, Konrad-Lorenz Str 24, 3430 Tulln, Austria; Department of Soil Ecology, UFZ - Helmholtz Centre for Environmental Research, DE-06120, Halle/Saale, Germany; iDiv - German Centre for Integrative Biodiversity Research, Halle Jena Leipzig, DE-04103, Leipzig, Germany; Department of Community Ecology, UFZ - Helmholtz Centre for Environmental Research, 06120 Halle/Saale, Germany

**Keywords:** Oak, Transcriptome, *de novo* assembly, Comparative genomics, RNA-seq, Bud phenology

## Abstract

**Background:**

Many northern-hemisphere forests are dominated by oaks. These species extend over diverse environmental conditions and are thus interesting models for studies of plant adaptation and speciation. The genomic toolbox is an important asset for exploring the functional variation associated with natural selection.

**Results:**

The assembly of previously available and newly developed long and short sequence reads for two sympatric oak species, *Quercus robur* and *Quercus petraea*, generated a comprehensive catalog of transcripts for oak. The functional annotation of 91 k contigs demonstrated the presence of a large proportion of plant genes in this unigene set. Comparisons with SwissProt accessions and five plant gene models revealed orthologous relationships, making it possible to decipher the evolution of the oak genome. In particular, it was possible to align 9.5 thousand oak coding sequences with the equivalent sequences on peach chromosomes. Finally, RNA-seq data shed new light on the gene networks underlying vegetative bud dormancy release, a key stage in development allowing plants to adapt their phenology to the environment.

**Conclusion:**

In addition to providing a vast array of expressed genes, this study generated essential information about oak genome evolution and the regulation of genes associated with vegetative bud phenology, an important adaptive traits in trees. This resource contributes to the annotation of the oak genome sequence and will provide support for forward genetics approaches aiming to link genotypes with adaptive phenotypes.

**Electronic supplementary material:**

The online version of this article (doi:10.1186/s12864-015-1331-9) contains supplementary material, which is available to authorized users.

## Background

Many northern-hemisphere forests are dominated by evergreen and deciduous oaks (*Quercus* spp.). The genus *Quercus* consists of about 400 species extending over a wide range of environmental conditions, from temperate to subtropical regions. Some sympatric species (such as *Q. robur*, *Q. petraea*, *Q. pyrenaica*, *Q. faginea*, and *Q. pubescens* in Europe) occupy different ecological niches [1] and are therefore interesting models for studies of plant adaptation [2] and ecological speciation [3]. An important question in biological science concerns the response of these long-lived organisms to rapid environmental change, their ability to evolve and the mechanisms involved. The genes and associated structural and expressional variants required for adaptation must be identified if we are to address these questions. To this end, a number of genomic tools and resources have been developed for oaks (reviewed in [4]), including two bacterial artificial chromosome (BAC) libraries [5], a large number of SSRs [6] that have been used to generate linkage maps [7] and expressed sequence tags (ESTs), mostly obtained by Sanger and Roche 454 sequencing [8,9]. Researchers can now use these tools to address concerns about the adaptability of forest trees at the genomic level. However, studies aiming to address this objective have been hampered by a lack of genomic resources. Ultra-deep sequencing methods, in particular, could help to expand the oak transcript catalog for studies of the genomic mechanisms underlying plastic responses and evolutionary adaptation to environmental change. RNA-seq is a method of choice for quantifying gene expression [10,11], and for identifying genes preferentially expressed at specific developmental stages [11] or in specific physiological conditions [12]. RNA-seq can be used to infer gene regulatory networks on the basis of enrichment analysis for pathways and gene ontology groups [13], using established knowledge from model organisms [14], or with dedicated statistical approaches [15] for the *de novo* identification of sets of co-expressed genes. In this study, RNAseq was used to identify genes regulated during bud dormancy release, an important phase of vegetative bud phenology, known to be strongly affected by temperature and photoperiod and therefore, likely to be greatly disturbed by the unprecedented warming associated with climate change [16]. Low temperatures are essential to overcome endo-dormancy (chilling requirement), but high temperatures are also required for bud break (heat requirement). The effect of climate change, with milder autumns and warmer winters, on the timing of bud flush and the impact of exposure to late spring frost are key questions in forestry requiring a detailed understanding of the physiological and molecular mechanisms (and their genetic variability) involved in dormancy release. We addressed these questions, by studying the dynamics of gene expression over this critical period, focusing on two successive phases of bud dormancy release: i) eco-dormancy, a dormancy state prevailing in late winter and spring imposed by environmental conditions unfavorable for growth (*i.e*. heat requirement not fulfilled), and ii) swelling bud, which occurs in spring, just before bud burst, when the heat requirement for bud break is almost satisfied.

Once established, transcriptome analysis can also be used in a comparative framework, to reveal some of the evolutionary features of a genome, through the inference of whole-genome duplication and speciation events, for example [17,18]. It has been proposed that modern eudicots have derived from a founder ancestral genome structured in 21 protochromosomes followed by series of whole genome duplications (WGD) or polyploidizations and ancestral chromosome fusions and fissions [19]. Polyploidization has been proposed as a key evolutionary mechanism in providing new genetic material leading to morphological and phenotypic innovations through neo and/or subfunctionalizations of duplicated gene pairs [19]. To this regards, how the twelve modern oak chromosomes evolved from the eurosid ancestor with respect to duplication and chromosome rearrangement patterns is still largely unknown. In this context, the main objectives of this study were: i) to enlarge the current oak EST resource through the use of ultrahigh-throughput sequencing technology and to combine the data obtained with available sequences expressed in different tissues, at different developmental stages, and in response to different biotic and abiotic stresses, to generate the most comprehensive annotated unigene set for oak, and ii) to use this resource to increase our understanding of the structure, function (focusing particularly on bud dormancy release) and macroevolution of the oak genome.

## Results

### Sequencing and assembly of the oak transcriptome

**Figure 1:**
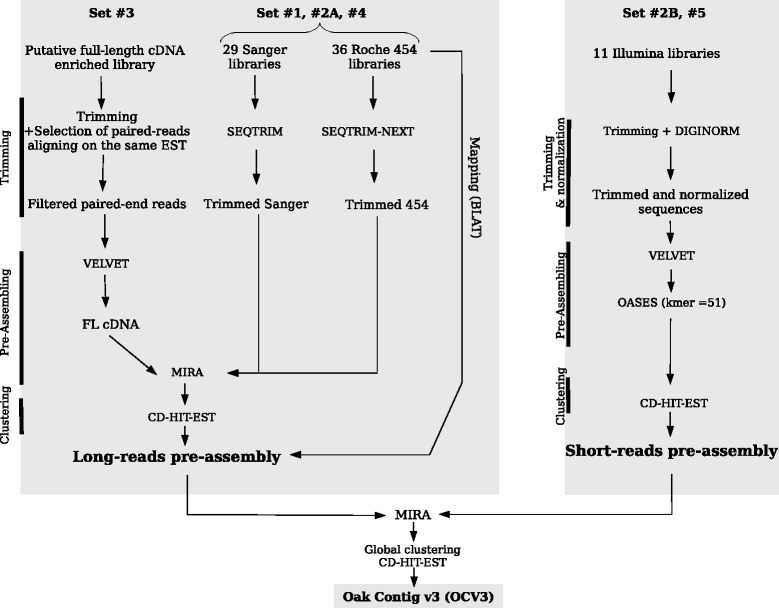
**Schematic representation of the bioinformatic analysis.**

Transcriptomes are a valuable genomic resource for studies in non-model organisms for which genome sequences are not available, because they are smaller and less complex than genomes. The *de novo* assembly of transcriptome sequence data from a single sequencing platform has become a routine task, and a handful of *de novo* transcriptome assemblers have been developed [20], but combining the outputs from multiple sequencing platforms remains challenging [21] and involves the use of suitable assembler software for different types of datasets (short/long; single/paired-end reads). In this study, we used a combination of Sanger, Roche-454 and Illumina technologies and bioinformatic tools to generate a catalog of oak transcripts from RNA obtained from different tissues, developmental stages and in response to biotic and abiotic stresses (Additional file 1). Long and short reads were assembled independently, with robust assemblers (see workflow in Figure 1 and detailed procedure in Additional file 2) and the resulting assemblies were combined to produce a final meta-assembly (Oak Contig V3.0, OCV3). The main characteristics of these two pre-assemblies and the final meta-assembly are summarized in Table 1A.

**Table 1:** **Description of oak transcriptomic assemblies**

**A/ Assembly**			**Long-reads**	**Short-reads**	**OCV3**
Number of trimmed sequences considered in the assembly			2,888,537	417,337,626	/
Number of trimmed sequences incorporated in the assembly			2,003,295	417,337,626	274,867
Number of contigs > 100bp, after redundancy reduction			44,272	230,595	192,097
Number of singletons			300,373	40,119,145	/
Consensus (total bp)			/	/	199,278,344
Average contig size (bp)			937	877	1,037
**B/**			**OCV1**	**OCV2**	**OCV3**
Number of contigs			69,154	65,712	192,097
Mean length (bp)			705	1,003	1,037
Median (bp)			606	734	597
N50 (bp)			908	1,545	1,879
Consensus (bp)			48,751,826	65,913,455	199,278,344
Nb of annotated contigs in SwissProt			34,614	38,292	63,857
Nb of unique SwissProt ID			13,333	16,429	17,476
**C/**	**Number of contigs > 100bp, after redundancy reduction**	**Assembly (total bp)**	**Mean contig size (bp)**	**Median contig size (bp)**	**N50 (bp)**
OCV3-91k	90,786	148,088,893	1,631	1,292	2,329
OCV3-101k	101,311	51,187,294	505	328	697
OCV3	192,097	199,278,344	1,037	597	1,879

**A** Characteristics of the long-reads, short-reads and meta assemblies (OCV3).

**B** Side-by-side comparison of oak unigene sets (OCV1: assembly from Ueno *et al.* [8], OCV2: assembly from Tarkka *et al.* [22], and OCV3: this paper). N50 length is defined as the length for which the collection of all contigs of that length or longer contains at least half of the total of the lengths of the contigs.

**C** Comparison between OCV3-91k (Unigenes with BlastX hit) and OCV3-101k (Unigenes without BlastX hit) subsets: assembled sequences (in bp), mean and median contig sizes, N50.

#### Long-read pre-assembly

The sequencing of 29 and 36 cDNA libraries with Sanger and 454 technologies (sets #1, #2A and #4 in Figure 1) resulted in 94,174 and 2,790,004 trimmed reads respectively. The distribution of trimmed ESTs is shown in Additional file 3. A total of 6,571 putative FL-cDNA clones (set #3) sequenced with the Illumina/Solexa GA-II X (PGTB, Plateforme Génome transcriptome de Bordeaux), yielded 17,196,106 paired end reads. Then, *de novo* assembly with Velvet and TGICL software yielded 4,359 contigs. By combining Sanger, Roche-454 and reconstructed FL-cDNA data, we obtained 2,888,537 long sequences used to construct a long-read pre-assembly with MIRA. We finally obtained 44,272 contigs, with a mean sequence length of 937 bp (standard deviation: 521 bp; N50: 1,118 bp, defined as the largest entity E such that at least half of the total size of the entities is contained in entities larger than E; see the black curve in Additional file 4).

#### Short-read pre-assembly

A total of 961,151,725 Illumina reads (set #2B and #5), 80.2% corresponding to Illumina sequences generated in the present study, allowed to generate a short-read pre-assembly of 230,595 contigs with a mean sequence length of 877 bp (SD 1,069 bp; N50 1,758 bp, red curve in Additional file 4).

#### Meta-assembly

The meta-assembly was generated with MIRA. Using the 274,867 pre-assembly contigs (44,272 long-read contigs and 230,595 short-read contigs), we obtained a final assembly (OCV3) consisting of 192,097 contigs, which is available from the Quercus portal (https://w3.pierroton.inra.fr/QuercusPortal/index.php?p=est). In total, 1,623 (0.84%) and 2,747 (1.43%) contigs yielded significant hits with the oak chloroplast and mitochondrial genomes, respectively. The mean contig size for OCV3 was 1,037 bp (SD 1,150 bp; N50 1,879 bp, green curve in Additional file 4) which is close to the mean gene length in eukaryotes (1,346 bp, [23]). By assembling short and long reads together in a single unigene set, we were able to improve the first oak transcriptome assembly (OCV1) established by Ueno *et al*. 2010 [8] from Sanger and Roche-454 reads (Table 1B). Simultaneously, we improved OCV2 recently established for a single *Q. robur* genotype (Table 1B, [22]). It is difficult to compare the size of the meta-assembly (about 192 thousand contigs) with those of other projects with similar aims and approaches, because it is influenced by genome and transcriptome sizes, the diversity of tissues/developmental stages/environmental conditions, the number of cDNA sequences produced, and the assembly method used. However, if we consider recent studies on forest trees, the OCV3 meta-assembly is of a similar size to those of *Pseudotsuga menziesii* (170,859 contigs [24]) and *Pinus pinaster* (210,513 contigs [25]), larger than that of *Castanea molissima* (40,039 contigs [26]), and smaller than of *Pinus contorta* (303,450 contigs, [27]).

### Functional annotation

#### Sequence similarity to SwissProt accessions and other plant proteomes

We characterized the oak transcriptome by a similarity-based approach, using proteomes of closely related plant species and SwissProt accessions. We used the BlastX algorithm to align the 192,097 OCV3 contigs with these proteomes, and obtained a significant match for 90,786 oak contigs (referred to hereafter as the OCV3-91 k subset). Similar numbers of hits were obtained with the selected gene models: 77,784 hits in *Arabidopsis thaliana* (*At*), the species most phylogenetically distant from *Quercus* considered, to 84,852 hits in *Prunus persica*, the closest species considered (Table 2). OCV3 contigs matched a total of 17,476 different SwissProt accessions and between 16,573 (*Vitis vinifera*) and 23,053 (*Populus trichocarpa*) sequences in plant gene models. The number of oak contigs displaying similarity in terms of deduced amino-acid sequences with the content of at least one of the databases studied (90,786) was much greater than the number of genes present in oak (about 30,000, as estimated from BAC-end sequences, [5]). This overestimation may be due to contig redundancy, contig fragmentation (contig breaks in low-coverage regions), unassembled alleles, particularly for highly polymorphic diploid species of this type, with a mean of one SNP or Indel every 25–30 bp [28], splicing variants, sequencing errors, or sequence read misattribution between closely related paralogs due to the presence of recently duplicated genes. We found that the 18,587 *Prunus persica* gene model sequences with a hit in OCV3-91 k matched, on average, 4.6 oak contigs each, highlighting the redundant nature of OCV3-91 k. However, we also found that 13,536 (i.e. 72.8%) of the matched *Prunus* gene model sequences displayed 75% coverage with a single oak contig each, indicating that many of the oak genes for which a closely related gene was present in peach were well assembled. However, paralog assembly may also have contributed to erroneous gene predictions. In the species with the closest phylogenetic relationship to *Quercus* analyzed here (i.e. *Prunus persica*) more than 19,000 different genes were tagged, corresponding to about two thirds of the protein-coding genes of oak.

**Table 2:** **BlastX results for OCV3 contigs against SwissProt database and the proteomes of five species**: ***Prunus persica***, ***Vitis vinifera***, ***Populus trichocarpa***, ***Eucalyptus grandis***, ***Arabidopsis thaliana***

	***Prunus***	***Vitis***	***Populus***	***Eucalyptus***	***Arabidopsis***	**SwissProt**	**All**
Nb of oak contigs with a hit	84,852	82,655	81,849	78,143	77,784	63,857	90,786
Nb of proteins with a hit	18,587	16,573	23,053	22,338	18,661	17,476	/
total nb of proteins or accessions	28,701	26,346	45,033	46,315	35,386	540,732	/

The remaining 101,311 contigs without BlastX hits in SwissProt or the selected plant gene models (referred to hereafter as the OCV3-101 k subset) were then aligned (using BlastX: e-value 1e^−5^ -E 2 -W 5) with the *nr protein* database and the genome sequence of *Prunus persica* (using BLAT and EST2 genome software, [29]). Only 2.4% of these contigs could be aligned with sequences in the *nr protein* database. These contigs also diverged much more than the OCV3-91 k subset (Table 3A) from the *Prunus persica* genome. Matches to the *Prunus persica* genome sequence were obtained for 59% of the OCV3-91 k contigs but only 6.2% of the OCV3-101 k contigs. Moreover, we successfully mapped 64,001 OCV3-91 k and only 8,380 OCV3-101 k contigs onto 17,038 and 5,265 *Prunus persica* gene models, respectively (Table 3B). The number of exons per gene model was three times higher for OCV3-91 k than for OCV3-101 k. All together, these results indicate that OCV3-101 k consisted mostly of non-coding RNA. Besides, OCV3-91 k contained 46,415 (51%) contigs supported by short reads only, whereas OCV3-101 k contained 86,575 (85%) such contigs. The shorter mean size of contigs in OCV3-101 k may also have resulted in the presence of less biological meaningful information. Indeed, mean and median contig sizes were three times greater in OCV3-91 k than in OCV3-101 k (Table 1C), consistent with the presence of a larger amount of valuable information for functional characterization of the oak transcriptome in OCV3-91 k. We therefore concentrated on OCV3-91 k for subsequent analyses.

**Table 3:** **Mapping results against the** ***Prunus persica*** **genome and gene models**

**A**	**OCV3**	**(OCV3-91k)**	**(OCV3-101k)**
Number of sequences	192,097	90,786	101,311
Number of mapped sequences	59,851 (31.1%)	53,600(59%)	6,251 (6.2%)
Number of matches	64,292	54,954	9,338
Number of matched exons	209,795	200,252	9,543
Number of exons/model	3.26	3.64	1.02
Mean of identity percent	83.99%	82.75%	91.26%
Number of monoexonics	29,767	20,591	9,176
**B**		**(OCV3-91k)**	**(OCV3-101k)**
Number of sequences		90,786	101,311
Number of mapped contigs		64,001 (70.5%)	8,380 (8.27%)
Number of *P. persica* gene models		17,038 (59.4%)	5,265 (18.3%)

**A** Mapping results (BLAT software) for OCV3, OCV3-91k (Unigenes with BlastX hit) and OCV3-101k (unigenes without BlastX hit) against the *Prunus persica* genome.

**B** Mapping results (BlastN) for OCV3-91k and OCV3-101k against the *Prunus persica* gene models.

#### Functional annotation and GO classification of oak transcripts

**Figure 2:**
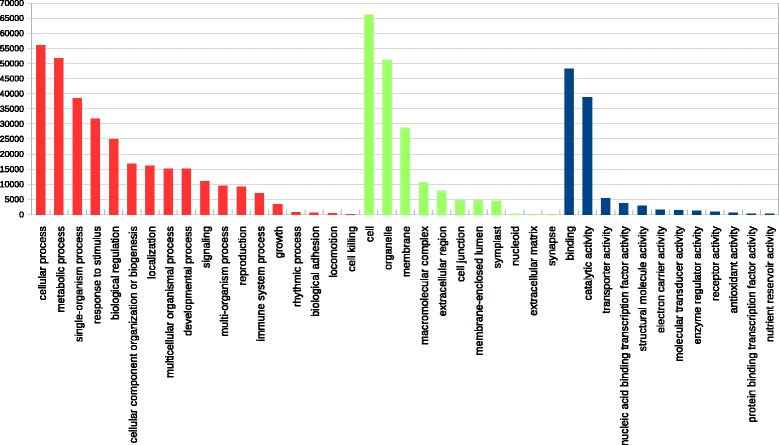
**Gene ontology classification of OCV3**-**91 k contigs.** GO Slim terms contributing to the annotation of the 77,277 OCV3_91k contigs. Red: Biological process. Green: Cellular component. Blue: Molecular function.

We assigned functions to the OCV3-91 k contigs with the Gene Ontology (GO) classification, which provides a standardized set of terms to describe the genes and gene products of different species. First, we designated functions for each contigs on the basis of matches with the Pfam database. A total of 1,112 GO terms to 24,999 contigs (*i.e*. 27.54%) were identified. A second series of GO annotation was based on the GOA database. For the 77,784 OCV3-91 k contigs giving significant matches to *A. thaliana* proteins, 65,198 (*i.e*. 71.82%) were annotated with at least one GO term. We retrieved the functional categories associated with their best Blast hits in SwissProt, and this yielded 13,355 GO terms for 61,139 contigs. Based on best Blast hit results, we were able to associate 76,457 contigs with GO terms in this second round. Overall, at least one GO term was assigned to 77,277 contigs (*i.e*. 85.12% of OCV3-91 k) (Additional file 5); 24,180 of these contigs (31.29%) were associated with GO terms from both series, 819 contigs (1.06%) were associated with GO terms from the first series, and 52,278 contigs (67.65%) were associated with GO terms from the second series. For the 77,277 contigs associated with at least one GO term, 72,558 were associated with 1,820 GO terms (70%) for Biological Processes (BP) , 72,235 were associated with 267 GO terms (10%) for Cellular Components (CC) and 71,458 were associated with 515 GO terms (20%) for Molecular Functions (MF) , accounting for a total of 2,602 GO terms (Additional file 6). These proportions are similar to those in the GOA database: 25,725 (66%), 3,474 (9%) and 9,685 (25%) GO terms for BP, CC and MF, respectively. Setting the Gene Ontology graph level to 2 decreased the number of GO terms considerably, to 18 for BP, 11 for CC and 12 for MF (Figure 2). The most abundant GO Slim terms were cellular process (GO: 0009987; 56,132 contigs), binding (GO:0005488; 48,227 contigs) and cell (GO:0005623; 66,082 contigs), for BP, MF and CC, respectively. Comparing the ranking of the main GO terms with those for other phylogenetically related species (e.g. *Castanea dentata*) and more distantly related species (e.g. *Eucalyptus grandis*, which is also a eurosids, and *Pseudotsuga menziesii*, from the Pinaceae), a remarkable match was found for the GO category Molecular Functions, for which sufficient comparable data were available for the four species (Additional file 7). These results suggest that a large proportion of the plant’s genes were present in OCV3-91 k. Finally, it would be tempting to highlight the differences in functional classes between the OCV3-91 K sequences associated with GO terms and *At* GO annotations. However, we believe that such an analysis would be misleading, given the complex nature of any transcript catalog. We will wait until the generation of an oak gene model before carrying out such analyses, to avoid making erroneous predictions [30].

### Comparative genomics and the detection of conserved orthologous sequences common to peach

**Figure 3:**
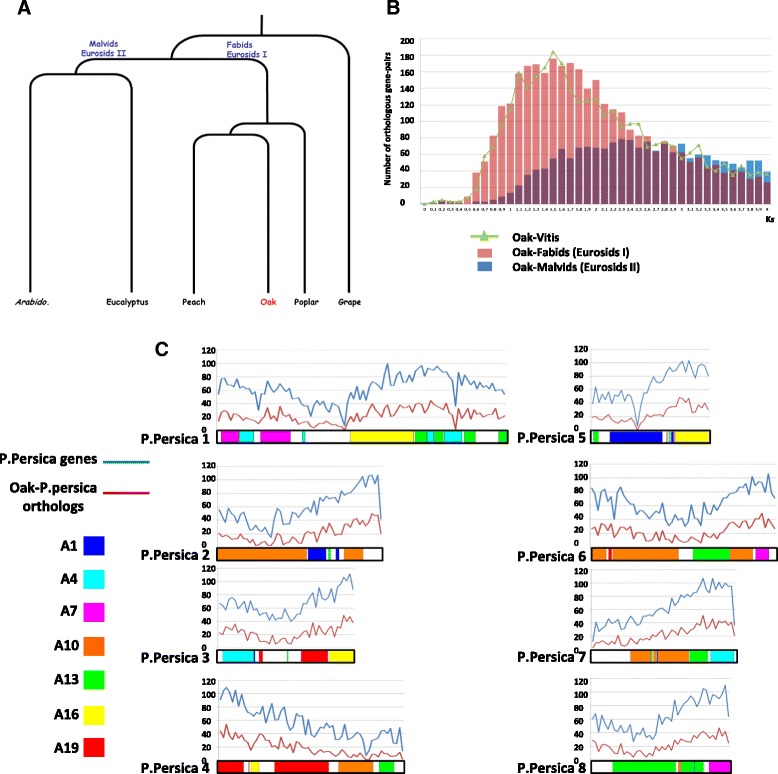
**Oak genome orthologous relationships. A**. Simplified phylogenic tree placing oaks among sequenced malvid (*Arabidopsis*, eucalyptus), fabids (peach, poplar) and basal rosid (grape) genomes. **B**. Distribution of K*s* values (*x*-axis) for orthologous gene pairs between oak and malvids (*Arabidopsis*, eucalyptus: in blue), between oak and fabids (peach, poplar: in red) and between oak and grape (in green). Grape is considered to be the modern representative of the ancestral eudicot genome. **C**. Distribution of peach gene models (blue curve) and 9,549 orthologous gene pairs with oak (red curve) along the 8 peach chromosomes. Peach chromosomes are illustrated as a mosaic of 7 colours highlighting the features of the ancestral eudicot chromosome (A1, A4, A7, A10, A13, A16, A19) as in [19].

The rosids comprise two major clades of orders [31]: the fabids (i.e. eurosids I) and the malvids (i.e. eurosids II). *Quercus* species are fabids and are relatively closely related to *Prunus* (Figure 3A). BlastX alignment of the OCV3-91 k contigs with the malvid (*Arabidopsis*, *Eucalyptus*), fabid (peach, poplar) and basal rosid (grape) gene models (Table 2) demonstrated that evolutionary relationships (based on the Ks metric, see the Methods section) between oak and fabid representatives (red distribution in Figure 3B) were stronger than those between oak and malvid representatives (blue distribution in Figure 3B). In particular, 62,593 OCV-91 k contigs yielded BlastX hits with *Prunus persica* (closely related to oak) model genes, 9,549 of which (corresponding to 60,189 oak contigs) were located on eight major scaffolds corresponding to the eight chromosomes of the peach genome. These 9,549 orthologous sequences (listed in Additional file 8) delivered the following chromosomal relationship density: chromosome #1: 5,910 peach genes (blue curve in Figure 3C)/2,104 oak orthologs (red curve), chromosome #2: 3,162/1,096, chromosomes #3: 2,932/1,001, chromosome #4: 3,373/1,128, chromosome #5: 2,469/883, chromosome #6: 3,685/1304, chromosome #7: 2,884/1,054, chromosome #8: 2,841/979.

This similarity in genome organization between oak and peach was used as an input to highlight the collinearity between the oak and peach genomes and to investigate whether genetic information from one species can be transferred to another, particularly as concerns phenology-related QTLs. In particular, further genetic mapping of the 9,549 characterized COS markers between oak and peach will immediately illuminate the synteny relationships with the malvids and fabids and ultimately evolutionary history of the 12 oak chromosomes from the eudicot ancestral genome reported as structured in 7 protochromosomes. Finally the delivered heterologous oak/peach map offer the opportunity to select oak genes either covering the entire peach genome or specifically located in a peach locus of interest related to a specific agronomic trait.

### Expression pattern for oak transcripts

This analysis was based on the high-throughput sequencing (Illumina technology) of six cDNA libraries (listed in Additional file 1): ecodB, eco-dormant bud; swB, swelling bud; XY, secondary differentiating xylem; RO, root; LE, leaf and CA, *in vitro* dedifferentiated callus. Two approaches were used to determine the tissue-related expression of oak contigs. First, the contigs were classified according to the tissue of origin of their reads, and the tissues were clustered on the basis of their expression profiles. Second, statistical tests were used to identify genes displaying significant differential expression between pairs of tissues. For biological interpretation, we focused on the comparison between two developmental stages of vegetative bud dormancy release: eco-dormancy (ecodB) and swelling bud before bud break (swB) (Table 4). The reference for this analysis was the OCV3-91 k subset.

**Table 4:** **Subset of genes differentially expressed between Ecodormancy and Swelling buds stages**

**Biological process**	**Gene function**	**Ath accession number**	**Fold change ratio** **(** **Ecodormant bud** **/** **Swelling buds** **)**	**position in the functional gene network**
**Genes up**-**regulated in Ecodormant buds**				
Ribosome biogenesis	T13C7.4 (60S ribosomal protein L14)	AT2G20450	>100	
Ribosome biogenesis	F12L6.5 (ribosomal protein L23A)	AT2G39460	>100	
Ribosome biogenesis	T9J14.13 (ribosomal protei S24e)	AT3G04920	>100	
Ribosome biogenesis	Ribosomal protein L232A	AT3G55280	>100	
Ribosome biogenesis	T25K17.40 (ribosomal protein L31e)	AT4G26230	>100	
Ribosome biogenesis	K16F13.2 (40S ribosomal protein S27-3)	AT5G47930	>100	
Ribosome biogenesis	MUP24.13 (60S ribosomal protein L12)	AT5G60670	>100	
Ribosome biogenesis	F10K1.22 (60S ribosomal protein L35a)	AT1G07070	>100	
Ribosome biogenesis	T2P11.7 (60S ribosomal protein L34)	AT1G26880	>100	
Ribosome biogenesis	F19K6.12 (60S ribosomal protein L37)	AT1G52300	>100	
Ribosome biogenesis	STV1 (ribosomal protein L24)	AT3G53020	>100	
Ribosome biogenesis	Zinc-binding ribosomal protein	AT3G60245	>100	
Ribosome biogenesis	PRPL11 (plastid ribosomal proteinL11)	AT1G32990	>100	
Ubiquitin dependent rotein catabolic process	UBC28 (ubiquitin conjugating enzyme 28)	AT1G64230	>100	Neighbors of sbi-miR169r-3p_agpf_35
Ubiquitin dependent protein catabolic process	FKF1 (flavin-binding kelch repeat F box 1)	AT1G68050	>100	Neighbors of ELF3, Neighbors of GI
Ubiquitin dependent protein catabolic process	UBQ11 (ubiquitin 11)	AT4G05050	>100	Neighbors of heat shock
Ubiquitin dependent protein catabolic process	ASK2 (Arabidopsis SKP-Like2)	AT5G42190	>100	
Ubiquitin dependent protein catabolic process	ATUBA1 (ubiquitin activating enzyme 1)	AT2G30110	>100	
Response to cold	DREB1A (DREB subfamily A-1)	AT4G25480	>100	Neighbors of cold stress, DREB and CBF
Response to cold	CBF1 (C repeat/DRE binding factor 1)	AT4G25490	>100	Neighbors of cold stress, DREB and CBF
Response to cold	1 (low expression of osmotically responsive gene	AT1G56070	>100	Neighbors of cold stress
Response to cold	LTI30 (Low temperature induce temperature)	AT3G50970	>30	Neighbors of cold stres, ABA and CBF
Response to cold	RCI3 (rare cold inducible gene 3)	AT1G05260	>100	Neighbors of cold stress
Response to cold	Fib (Fibbrilin 1A)	AT4G04020	>50	Neighbors of ABA
Response to water deprivation	ATBI-1 (Bax inhibitor 1)	AT5G47120	>100	Neighbors of drought and COLI
Response to water deprivation	SIP3 (CBL interacting protein kinase 6)	AT4G30960	>100	
Response to water deprivation	CBL9 (calcineurin B like protein 9)	AT5G47100	>100	Neighbors of ABA, drought and cold
Response to gibberelin stimulus	Gasa1 (GAST1 protein homolg1)	AT1G75750	>100	
Response to gibberelin stimulus	Gasa2 (GAST1 protein homolg2)	AT4G09610	>100	
Response to gibberelin stimulus	AGL20 (Agamous like 20)	AT2G45660	>100	Neighbors of AP1
Response to high light intensity	Bag6 (Bcl-2-associated athanogene 6)	AT2G46240	>100	Neighbors of heat shock
**Genes up**-**regulated in swelling buds**				
DNA dependent DNA replication initiation	MCM6	AT5G44635	>3	
DNA dependent DNA replication initiation	MCM3	AT5G46280	>3	
DNA dependent DNA replication initiation	PRL (prolifera)	AT4G02060	>2	Neighbors of cell cycle , DNA replication
DNA dependent DNA replication initiation	CDC45 (cell division cycle 45)	AT3G25100	>6	Neighbors of mitosis , DNA replication
DNA dependent DNA replication initiation	T12C22.19 (MCM2)	AT1G44900	>2	
Regulation of cell cycle and cell division	CYCB 1;4 (cyclin dependent protein kinase)	AT2G26760	>10	
Regulation of cell cycle and cell division	CYCD1;1 (cyclin D-type protein)	AT1G70210	>10	Neighbors of CYCD1;1 and CYCD1;3
Regulation of cell cycle and cell division	CYCD5;1 (cyclin D-type protein)	AT4G37630	>100	Neighbors of cell cycle
Regulation of cell cycle and cell division	CYCA3;2 (cyclin D-type protein)	AT1G47210	>2	Neighbors of morphogenesis and cell differenciation
Regulation of cell cycle and cell division	CYCD3;1 (cyclin D-type protein)	AT4G34160	>7	Neighbors of CYCD1;1 and CYCD1;3
Response to auxin	OBP1 (OBF binding protein)	AT3G50410	>3	Neighbors of cell cycle
Response to auxin	Aux1 (auxin influx transporter)	AT2G38120	>6	Neighbors of primordium elongation s and cell differenciation
Response to gibberellin	Gasa4 (Gast1 protein homolog 4)	AT5G15230	>10	Neighbors of heat shock and flower development
Response to gibberellin	Myb26 (MYB domain protein 26)	AT3G13890	>100	Neighbors of cell development
Response to brassinosteroid	Bas1 (cythochrom P450)	AT2G26710	>7	Neighbors of leaf development and hormone
response to brassinosteroid	T5I8.2 (hercule receptor protein kinase 2)	AT1G30570	>40	Neighbors of brassinolide
Response to sucrose stimulation	GBF6 (leucine zipper11)	AT4G34590	>2	
Response to sucrose stimulation	GASA6 (GA stimulated arabidopsis 6)	AT1G74670	>10	

The most differentially expressed GO terms identified in the enrichment analysis are indicated in the first column for each dormancy stage.

The localization of the genes in the functional network is indicated in the last column when available.

#### Identification of transcripts differentially expressed across a panel of tissues

An inventory of the numbers of contigs present in several oak tissues or specific to a given tissue revealed that RO (roots harvested from six-month-old seedlings after exposure to cold, heat, high CO_2_ concentration, water stress and hypoxia) made the greatest contribution to the OCV3-91 k contigs, with 78,502 (86.47%) matching contigs (Additional file 9). Adding a second tissue (endodB) to the RO dataset further increased the number of contigs by 6.7% (6,084 new contigs). Successive additional inclusions of LE, CA, swB and XY increased the number of contigs by 2.43% (2,202 contigs), 1.01% (919 contigs), 0.57% (521 contigs) and 0.31% (283 contigs), respectively. We found that 56,672 contigs (62.42%) contained reads from all six tissues. These 56,672 contigs were particularly long, with a mean length of 2,025 bp. We also found that 66,885, 73,305, 78,480 and 83,601 contigs contained reads from at least 5, 4, 3, and 2 tissues, respectively. Finally, in total, 4,910 contigs were associated with a single tissue type: 1,756 were specific to RO, 1,039 to ecoDB, 977 to LE, 514 to CA, 341 to swB and 283 to XY). The mean length of these contigs was 684 bp (Additional file 10). As expected, a large part of the transcriptome is shared by all tissues. Nevertheless, sequencing of diverse tissues allowed to identify transcripts specific to each of them and was required for comparing expression level of genes involved in dormancy. The list of “tissue-specific” transcripts, with annotations, is provided in Additional file 11. These “tissue-specific” transcriptomes yielded valuable and specific additive information for inclusion in the catalog of oak transcripts, which can be accessed by interested scientists. Normalized read counts are provided for OCV3-91 K and OCV3-101 K in Additional file 12.

Tissues were then clustered according to their transcriptomic distances, based on the 91 k annotated contigs. Two major groups were identified on the resulting dendrogram, shown in Additional file 13. The first cluster included tissues resulting from primary (bud) and secondary (xylem) meristem activities. Interestingly, encodB clustered more closely with XY than swB, suggesting that very different regulatory networks control these two phenological phases of bud dormancy release. In the second cluster the two highly specialized tissues, RO and LE, clustered closer to each other than to CA, probably due to the very specific nature of the totipotent state of the *in vitro* dedifferentiated callus tissue.

**Figure 4:**
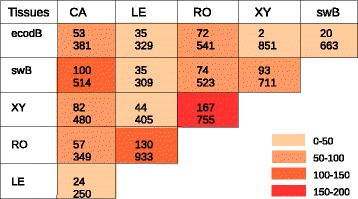
**OCV3-91 k contigs differentially expressed between six pairs of tissues (ecodB: ecodormant bud, swB: swelling bud, XY: differentiating secondary xylem, RO: root, LE: leaf and CA: dedifferentiated** ***in vitro*** **callus).** Number of significantly differentially expressed contigs identified by three (upper number) and two (lower number) statistical methods.

We used three methods to identify contigs displaying differential expression between each pair of tissues: R statistics, EdgeR, and DESeq (see the Methods section). RO and XY were ranked first in terms of the number of contigs with expression levels different from those in other tissues (Figure 4), whereas ecodB showed the lowest level of differential expression, particularly when compared with swB and XY, consistent with the clustering result. The results for *in vitro* callus tissue (totipotent state) were not consistent with our initial expectations, *i.e*. the expression of a much larger array of “specific” genes than the more specialized tissues, such as root, leaf or xylem.

#### Identification of candidate genes for differential expression during bud dormancy release

**Figure 5:**
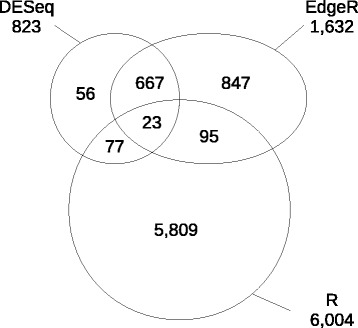
**Venn diagram showing the number of differentially expressed genes during bud dormancy release identified by 3 statistical algorithms; 6,004 differentially expressed contigs were identified by the R statistics method, 823 by DESeq and 1,632 by EdgeR (see** **Materials and Methods** **).**

The mapping of the 59,050,722 ecodB and 63,191,029 swB paired-reads onto the OCV3 assembly was successful for 21,137,289 ecodB reads and 23,699,876 swB reads. The ecodB and swB reads were integrated into 153,783 OCV3 contigs. The ecodB and swB reads were distributed between 136,441 and 134,875 contigs, respectively. Reads from both libraries were simultaneously detected in 117,533 contigs, whereas 18,908 contigs contained reads from ecodB only and 17,342 contigs contained reads from swB only. Analysis of the 153,783 integrated contigs with R statistics (see the Methods section) identified 6,004 (3.13%) contigs displaying differential expression (R > 8), whereas the DESeq and EdgeR R Bioconductor packages detected 823 (0.43%) and 1,632 (0.85%) differentially expressed contigs, respectively (FDR 5%). Only 23 contigs were identified by all three methods (Additional file 14) and 862 contigs were identified by at least two statistical methods (Figure 5 and Additional file 15). In total, 663 of these 862 contigs belonged to OCV3-91 k. GO term enrichment analysis was performed for these 663 differentially expressed contigs, with Pathway Studio software. Both the Gene and Plant Ontology databases were used.

Among the 663 contigs, 340 were found to be upregulated in ecodormant buds (146 “specifically” at this phenological stage and 194 were more strongly expressed in ecodB than in swB, Additional file 16). For this first set of genes, enrichment analysis for pathways and groups (EAPG, *p*-value < 0.05) yielded 202, 71 and 137 hits for the biological process (BP), cellular component (CC) and molecular function (MF) categories of the Gene Ontology database, respectively (Additional file 16). The five BP terms displaying the highest level of enrichment corresponded to “ribosome biogenesis”, “translation”, “response to cold”, “response to water deprivation” and “response to cadmium ion”. Similar results were reported by Ueno *et al*. in 2013 [32] for 454 sequencing on eco-dormant buds. In this previous investigation, a forcing test was used to determine the dormancy status of the buds. No such test was carried out in our study. The similarity in BP terms between the two studies suggests that the buds analyzed here were also in the ecodormancy phase. The first five MF groups related to “structural constituent of ribosome”, “transmembrane receptor activity”, “calmodulin binding”, “translation factor activity”, “nucleic acid binding” and “nucleoside-triphosphate activity”. Finally, 86 significant hits (EAPG, *p* < 0.05) were obtained against the Plant Ontology database, with the ontologies displaying the highest levels of differential expression corresponding to “guard cell”, “stamen”, “LP.08 eight leaves visible”, “LP.06 six leaves visible” and “male gametophyte”.

A total of 323 contigs were upregulated in swelling buds, 44 being “specific” to this phenological stage and 279 being more strongly expressed in swB than in ecodB (Additional file 16). For this second set of genes, 192, 45 and 148 EAPG hits (*p* value < 0.05) were obtained with the BP, CC and MF terms of the Gene Ontology database, respectively. The five BP categories displaying the highest level of enrichment were “DNA-dependent DNA replication initiation”, “regulation of cell cycle”, “DNA unwinding involved in replication”, “cell cycle and microtubule-based movement activity”. The MF categories displaying the highest level of enrichment were “carboxylesterase activity”, “lipid binding”, “microtubule motor activity”, “DNA-dependent ATPase activity” and “cyclin-dependent protein kinase regulator activity”. Finally, 102 significant hits (EAPG; *p* < 0.05) were obtained against the Plant Ontology database, the BP categories displaying the highest level of enrichment being “IL.00 inflorescence just visible”, “pedicel”, “F mature embryo stage”, “expanded cotyledon stage” and “4 anthesis groups”.

**Figure 6:**
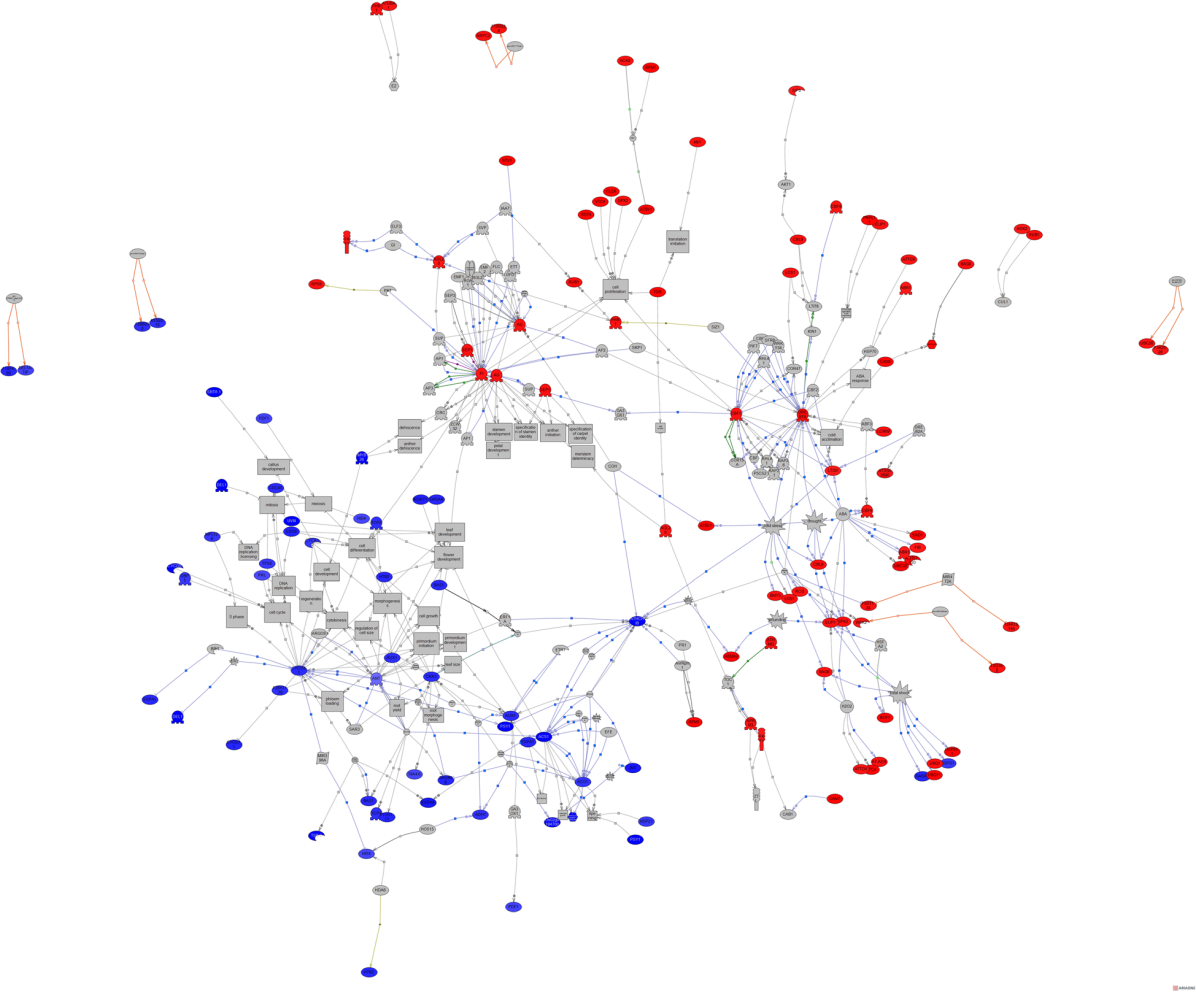
**Functional network predicted from the list of genes upregulated in ecodormant bud (in red) or swelling bud (in blue) based on the subset of 663 differentially expressed OCV3-91 k contigs (listed in Additional file** **16** **).** It is possible to zoom on particular parts of the network in the .tif file.

Subnetwork enrichment analysis (FNSE function in Pathway Studio; *p* < 0.05) was then performed for this set of 663 differentially expressed contigs (Additional file 16). Two distinct subnetworks were constructed from *Arabidopsis* homologs of ecodB and swB contigs with Pathway Studio; they were merged, as presented in Figure 6. Mean expression values shown in red (more strongly expressed in ecodB) and blue (more strongly expressed in swB) highlight the differences in central hubs and associated partners between these two phenological stages. EAPG (*p* < 0.05) on entities of these two subnetworks clearly supported our view that the gene expression patterns in eco-dormant and swelling buds were truly different. Indeed, the central hubs identified during ecodormancy related mostly to resistance to cold stress and water deprivation, whereas those identified in swelling buds related mostly to cell division and development (see discussion section).

## Discussion

The unigene set established in this study constitutes the most comprehensive transcript catalog assembled to date for the genus *Quercus*. We have also used this resource to design a high-density SNP array, and we have validated the SNPs detected *in silico*, by evaluated their Mendelian segregation in four pedigrees and determining their level of diversity in four white European oak species [33]. We discuss here the ways in which this resource improves our understanding of the molecular mechanisms involved in vegetative bud dormancy release, by comparing the abundance of mRNAs at two phenological stages: ecodormant buds and swelling buds just before bud break. This section therefore provides an overview of the molecular mechanisms involved in dormancy release in pedunculate oak in light of both the ontologies and central hubs identified in the enrichment analysis (Figure 6). Tissue specificity was also considered, in an attempt to identify specific markers of these two phenological stages.

### Transcripts upregulated during ecodormancy

In trees, ecodormancy occurs when unfavorable environmental conditions (mostly cold temperatures in temperate regions) prevent bud break in early spring. The molecular machinery involved in ecodormancy is poorly understood, but recent studies have reported an accumulation of transcripts relating to cold stress, water deprivation and hormonal stimuli [34].

#### Ribosome biogenesis

In total, 17 genes belonged to this functional category, a key component of the regulation of gene expression (Table 4). Thirteen of these genes were expressed in a bud-specific manner, but four were expressed in all the tissues studied. Most of the bud-specific genes encoded proteins very similar to ribosomal proteins (*AT2G20450*, *AT2G39460.1*, *AT3G04920*, *AT3G55280*, *AT4G26230*, *AT5G47930*, *AT5G60670*, *AT1G07070*, *AT1G26880*, *AT1G52300* and *AT3G53020*), but one (*AT3G60245*) encoded a zinc-binding ribosomal protein. In plants, exposure to low temperatures rapidly leads to major changes in the proteome, probably driven to some extent by changes in ribosome biosynthesis modulated by changes in levels of expression of the structural components of ribosomes. Degenhardt *et al*. [34] reported that the two paralogs of the *Arabidopsis thaliana RPL23* gene (also identified in this ontology) responded coordinately to developmental and stress stimuli, such as cold acclimation. A gene (AT1G32990) encoding a plastid ribosome protein (PRPL11) was also identified in this functional category. PRPs are major components of the plastid ribosome. In 2014, Song *et al*. [35] showed, in a rice mutant with PRP protein downregulated, that the accumulation of the corresponding transcript was strongly regulated by cold stress. They hypothesized that this gene was essential for normal chloroplast development during freezing tolerance.

In ecodormant buds, the cells have to deal with the instability of biomolecules. The synthesis of new ribosomes may facilitate maintenance of the translational machinery of the cell under unfavorable conditions. In the meantime, protein catabolism should eliminate malformed or non-functional proteins. We found significant enrichment for the term “ubiquitin-dependent protein catabolic process”, which was represented by five genes (“ubiquitin-dependent protein catabolic process”: *UBC28*, *FKF1*, *UBQ11*, *ASK2*, *ATUBA1*, Additional file 16).

#### Response to cold

In total, 15 genes belonged to this functional category, four of which were expressed specifically in buds, five of which were overexpressed in buds (fold-change ratio ≥10 with respect to the other tissues of the panel) and six of which were constitutively expressed (Table 4). Two relevant transcription factors for cold acclimation (*DREB1A* and *CBF1*) were identified in this study. These transcription factors are known to regulate the expression of many cold-responsive genes [36] promoting the initiation of cold acclimation and freezing tolerance in plants. Their overexpression in ecodormant buds may therefore increase the tolerance of meristematic cells to low temperature. A *Los1* gene encoding a translation elongation factor 2-like protein was also identified in this category. This gene has been strongly implicated in the development of freezing tolerance in *Arabidopsis thaliana*. Guo *et al*. [37] identified the *Los1* gene as a key regulator of the CBF1/DREB1 complex. Indeed, they showed that a lack of expression of this gene led to a lack of translation of the *CBF1* and *DREB1* transcripts, resulting in the repression of genes involved in cold acclimation. Several cold-responsive genes (members of the *LTI* and *RCI* gene families) were also identified, including *LTI30*, which encodes a dehydrin protein known to accumulate during cold stress. The expression of this gene is also tightly regulated by the *CBF transcription factors* [38] and ABA, an important hormone involved in seed dormancy. *RCI3*, which encodes a rare cold-inducible protein, was also identified. The precise role of *RCI* genes in dormancy regulation remains unclear, but several studies have reported the accumulation of transcripts from these genes in the bud during dormancy [39], suggesting a possible role in freezing tolerance. Finally, a *Fib* gene, encoding a fibrillin protein, was also identified. Fibrillins are lipid-binding proteins known to accumulate under cold stress. It is thought that they may be involved in the photoinhibition of PSII during cold stress [40], thereby protecting the chloroplast against frost damage. This gene is also regulated by the *CBF transcription factor* [41].

#### Response to water deprivation

Eight genes belonged to this functional category (Table 4). One was specifically expressed in the bud (*ATBI*-*1*), three were overexpressed in the bud (fold-change ratio > 10, *DREB1A*, *LTI 30* and *SIP 3*) and four were constitutively expressed in all the tissues of the panel. Some of these genes were also identified in the ontology terms corresponding to the response to cold stress, suggesting that some molecular functions are common to these two biological processes. *ATBI*-*1* encodes a Bax inhibitor 1 protein localized in the endoplasmic reticulum. Bax inhibitor genes were identified in both animals and plants. The precise molecular function of these genes is poorly understood, but they are probably involved in preventing the cell death induced by diverse biotic and abiotic stresses (reviewed by Ishikawa *et al*. [42]). This suggests that this gene may be involved in delaying cell death in the bud during cold stress, enabling the cell to cope with unfavorable environmental conditions. *SIP3* encodes CBL-interacting protein kinase 6. Once activated, the products of *CBL* genes transduce the calcium signal by phosphorylating downstream signaling components. He *et al*. [43] reported that the CBL-interacting protein kinase 6 of cotton played a role in the drought stress response, through regulation of the expression of targeted genes. *Arabidopsis* mutants constitutively expressing this gene are also characterized by an enhanced tolerance to drought and salt stress, suggesting a possible role of this transcription factor in adaptation to diverse abiotic stresses. Finally, *CBL9* encodes calcium sensor calcineurin B-like 9. Doğramaci *et al*. [44] reported the involvement of this calcium sensor protein as a key component of the ABA signaling pathway. Mutant line overexpressing this gene was found to be hypersensitive to ABA during seed germination and seedling growth.

#### Response to gibberellin stimuli

Five genes belonged to this functional category. Three genes were overexpressed in the bud (*T30D6.7*, *GASA1* and *GASA 3*, fold-change ratio from 3 to 10) and two were constitutively expressed (Table 4). Two of the genes identified (*GASA1* and *GASA3*) encode a GA-stimulated transcript (*GAST*) homolog. Da Silveira Falavigna *et al*. [39] reported the overexpression of *GAST* genes in the dormant buds of apple trees. A similar result was also reported by Doğramaci *et al*. [44] for leafy spurge. The precise role of *GAST* genes in dormancy regulation remains poorly understood, but these authors suggested a key role for gibberellin in dormancy regulation. The *AGL20* gene, another gene from this category, encoding a protein very similar to the AGAMOUS-LIKE 20 protein, was also identified. *AGL*-*20* is a *MADS box* gene that has been reported to encode an integrator of several environmental stimuli. Its level of expression is correlated with flowering time in *Arabidopsis thaliana* [45]. Trainin *et al*. [46] reported the possible involvement of polymorphism of this gene in the regulation of dormancy release in apricot, suggesting a possible role for *AGL20* in the breaking of dormancy.

#### Response to high light intensity

Three genes were identified in this category. One was specifically expressed in buds (*RPL23AB*, also involved in ribosome biosynthesis), one was overexpressed in bud (*BAG6*, fold-change ≥ 10) and one was constitutively expressed (*T1P17.2*). BAG proteins are much less well understood in plants than in animals. BAG proteins are characterized by a BAG domain that interacts with the ATPase domain of HSP 70/HSC70. In *Arabidopsis thaliana*, BAG proteins are encoded by an eight-member multigene family thought to be involved in programmed cell death through calcium signaling [47]. Kobayashi *et al*. [48] described a possible role for plant BAG proteins in floral transition, through activation of the expression of the *CONSTANS* gene. To our knowledge, our study is the first to report the overexpression of this gene during ecodormancy.

### Transcripts upregulated in the swelling bud

The mechanisms underlying bud break have been less thoroughly studied than those underlying ecodormancy and endodormancy. The ontology terms associated with this subset of genes correspond to cell division (Additional file 16), indicating a “restarting” of mitotic activity in the meristematic cells. This reinitiation of mitosis must occur before bud break, when environmental conditions become favorable.

#### DNA-dependent DNA replication initiation

Five genes belonged to this category (Table 4). None were specifically or preferentially expressed in the highly specialized tissues of the bud. Four of these genes (*AT5G44635*, *AT1G44900*, *AT5G46280* and *AT4G02060*) encode proteins very similar to minichromosome maintenance proteins (MCM proteins). In plants, MCM proteins have been implicated in cell division and are responsible for ensuring that the DNA of the cell is replicated only once per cell division. MCM proteins are encoded by a six-member multigene family and they interact with each other to form a complex. MCM proteins are relatively well characterized in plants. We identified *MCM6* (*AT5G44635*) in this study, a gene that has been reported to be essential for normal plant growth and development [49]. Dang *et al*. [50] showed that its expression was induced during salt and cold stress. This gene is also strongly expressed in active dividing tissue, suggesting a major effect of *MCM6* during cell cycle and proliferation [50]. A *Prolifera* gene (*PRL*) was also found to be upregulated. *PRL* also belongs to the *MCM* family and encodes an essential component of the DNA replication apparatus operating during the S-phase of the cell cycle. This gene is known to be strongly expressed during plant development. Springer *et al*. [51] reported this gene to be particularly strongly expressed in dividing cells during embryo development. They subsequently [52] showed that the *PRL* gene was also expressed in the cells responsible for initiating flower primordia. Finally, a *CDC45* gene from this category was also identified. Several authors have suggested that the product of the *CDC45* gene may function with the *MCM* complex, because several genetic and biochemical interactions between these components have been reported (reviewed by Steven *et al*. [53]). Other authors (e.g. Zou *et al*. [54]) have demonstrated particular interactions between this gene and the *MCM2* gene (also identified in our study, *T12C22.19*) in DNA elongation during the cell cycle. These findings are consistent with a strong reinitiation of mitotic activity in the meristematic cells of the swelling bud, enabling the bud to burst when environmental conditions become favorable.

#### Regulation of the cell cycle and cell division

This functional category was defined by merging two highly similar ontologies (the cell cycle and regulation of the cell cycle ontologies). It included 13 genes, but the redundancy rate was high because most of the cyclin (CYCB: cyclin-dependent protein kinase and CYCD: cyclin D-type protein) genes were present in both ontologies (Table 4). As for the previous ontology, no gene was found to be specifically and preferentially upregulated in buds. *CYCD* genes are known to be upregulated during the breaking of dormancy and their products act during the transition from G1- to S-phase in the cell cycle. In plants, cells in G1-phase expand and prepare for DNA replication, which occurs during S-phase, just before mitosis (G2-phase). The transition from G1- to S-phase is well understood in plants and several CYCD proteins have been identified in *Arabidopsis thaliana* (reviewed by Horvath *et al*. [55]). The CYCD proteins are also known to respond to various stimuli, including brassinosteroids and gibberelic acid (see the next section) or sugar (see the last section). In non-dormant cells, the product of the *CYCD* gene interacts with the cyclin-dependent protein kinase (*CDCB* genes also belong to this functional category) to form a complex. The formation of the CYCD/CYCB complex induces phosphorylation the retinoblastoma protein. This phosphorylation step triggers the release of *transcription factor*-*like EF2*, which induces the expression of a battery of genes essential for DNA biosynthesis, leading to transition from the S- to the G1-phase in the cell undergoing mitosis. The genes from this functional category identified in this study suggest that the genes involved in cell division are reactivated in swelling buds, to produce new cells and to prepare the bud for budburst.

#### Response to auxin, gibberellin and brassinosteroid stimuli

This category was obtained by merging three different ontologies (responses to gibberellin, auxin stimulus and brassinosteroid stimuli) (Table 4). Fifteen genes involved in hormone responses were identified in the GO terms enrichment analysis. Hormones are an essential component of dormancy regulation in perennial species. For example, Anderson *et al*. [56] reported involvement of an interaction between ABA and gibberellin in the loss of apical dominance. Gibberellins are also known to regulate several developmental processes, such as stem elongation, seed germination and dormancy. Five genes from the response to gibberellin stimulus ontology were found, including i) a GASA4-encoding protein. *GASA* genes are *gibberellin*-*responsive genes* involved in several developmental processes in plants. *GASA4* is expressed mostly in meristematic regions, consistent with a possible role in cell division [57]. Similar results were obtained for leafy spurge, in which the *GASA4* gene was found to be overexpressed in tissues undergoing active cellular division [58], and ii) a *MYB26* gene. The precise role of this transcription factor in the regulation of dormancy regulation has not been determined, but several authors (e.g. Skirycz *et al*. [59]) have reported an essential role for this gene in anther development and the regulation of its expression by both auxin and gibberellin.

Seven genes involved in the response to auxin stimulation were identified, including i) an *OBP1* gene highly similar to the gene encoding the DOF1 protein. In *Arabidopsis thaliana*, Skirycz *et al*. [59] reported the involvement of the DOF1 protein in the control of cell division and showed that the overexpression of the gene encoding this protein led to the upregulation of many cell-cycle genes. Using SSH hybridization technology, Derory *et al*. [60] also showed that some *DOF* genes were upregulated in sessile oak during bud burst, and ii) an *AUX1* gene. In *Arabidopsis thaliana*, *AUX1* belongs to a multigene family involved in regulating various auxin-dependent developmental processes, such as root gravitropic responses (reviewed by Péret *et al*. [61]). Other authors have reported upregulation of the *Aux1* gene during seed germination, in a mechanism comparable to dormancy release [62]. These findings suggest that plant hormones involved in swelling buds are essential for the regulation of cell division in meristematic cells.

Finally, three genes were identified in the response to brassinosteroid stimulus category. Again, none was specific to or preferentially expressed in the bud. These genes included: i) the *BAS1* gene encoding a member of the *cytochrome p450* family. *Arabidopsis* plants in which the *BAS1* gene is downregulated have a shorter hypocotyl, due to a phytochrome B defect. Neff *et al*. [63] showed that these mutants were also hypersensitive to brassinosteroids in a light-dependent manner, suggesting that the *BAS1* gene played an important role in connecting the photoreceptor and the brassinosteroid signaling pathway. Photoreceptors are essential for dormancy regulation. Indeed, several authors have shown that *phytochrome* and the *Constans* genes are essential components of the short-day signaling pathway during growth cessation (reviewed by Karlberg *et al*. [64]), and ii) a *T5I8.2* gene, similar to the *Hercule2* gene from *Arabidopsis thaliana. Hercule* genes encode receptor protein kinases from one of the largest known multigene families, with up to 600 members identified to date. *Hercule* genes are also known to be regulated by brassinosteroids. Riou-Khamlichi *et al*. [65] reported a possible role for some *Hercule* genes in regulating a battery of genes involved in plant growth and showed that *Hercule* genes were required for cell elongation during vegetative growth.

#### Response to sucrose stimulation

Three genes were identified in this category (Table 4). Sucrose appears to be a central molecular actor in the reinitiation of mitotic activity, as it is an essential component in the activities of the cell. Indeed, in perennial species, sucrose is the main source of carbon. Several authors have suggested that sucrose is a key factor involved in cell division and that there must be a specific mechanism for sensing cellular sugar levels in plants, to control the cell cycle [65]. It is well known that, during paradormancy, sugars are essential for expression of the *CYCD* genes (described above but also belonging to this category), which are involved in cell division (reviewed by Anderson *et al*. [56]). Among the genes from this category identified here was a *GBF6* gene very similar to a *basic leucine zipper 11* (*bZip11*) gene. Hanson *et al*. [66] reported that the translation of the *basic leucine zipper 11* gene transcript was strongly regulated by cellular sucrose concentration. Moreover, two key genes encoding enzymes involved in nitrogen metabolism (asparagine synthase and proline dehydrogenase) have been shown to be strongly regulated by the *Bzip11* transcription factor. Comparisons of ripened and dormant wheat seeds have shown an activation of nitrogen metabolism in the ripened seeds, suggesting a possible role of nitrogen metabolism in the recommencement of cell activity in pedunculate oak [67]. A *GASA6* gene was also identified. Gonzali *et al*. (2006) reported a downregulation of the *Arabidopsis thaliana GASA6* gene after sugar application [68]. However, there is currently no functional annotation for the *GASA6* gene, making it difficult to speculate on the function of the product of this gene in dormancy regulation.

## Conclusion

Oaks are cornerstone species with a fundamental role in temperate forest ecosystems. We therefore carried out a large-scale transcriptome analysis on two sympatric European white oaks. The resulting reference transcript catalog (OCV3), established with various actively growing tissues/organs, provides the most comprehensive survey of gene expression for the *Quercus* genus published to date. The information provided by this study already has proven useful, for the development of molecular markers for high-density linkage map construction and for studies of the degree and structure of genetic diversity in different oak species [33]. The regulation of some transcripts was found to be “tissue-specific”. These transcripts may therefore be considered good candidates for genes with specific functions in these tissues. In particular, the gene expression networks identified during vegetative bud release are of key importance as far as the seasonal growth of oaks is concerned, and are a valuable target for investigation in terms of the environmental changes resulting from global warming. A comparative analysis with *Prunus persica*, a phylogenetically related species, led to the detection and location of sequences orthologous to oak transcripts on peach chromosomes, providing relevant anchor points for further comparative genomics and genetic analyses of these two genera. Finally, this atlas will serve as a useful resource for annotating the reference genome sequence [30] and will provide support for forward genetics and population genomics approaches aiming to identify genes of importance for forest tree adaptation.

## Methods

### Plant material, library construction and sequencing

For establishment of the most comprehensive catalog of expressed genes in oak, we assembled cDNA sequences from five datasets (set #1-5 in Additional file 1 and Figure 1) into contigs:

- Set #1 was obtained from Ueno *et al*. [8] and consisted of 26 and 14 cDNA libraries from tissue panels for *Q. robur* and *Q. petraea*, sequenced by the Sanger and 454 methods, respectively,
- Set #2 consisted of: i) 16 normalized and 454 sequenced libraries from leaves pooled from various developmental stages, and roots (set #2A), with controls and treatments including the gypsy moth *Lymantria dispar*, powdery mildew *Erysiphe alphitoides*, oomycete root pathogen *Phytophthora quercina*, root nematode *Pratylenchus penetrans*, symbiotic fungus *Piloderma croceum*, mycorrhizal helper bacteria, *Streptomyces* sp. AcH 505 and the springtail *Protaphorura armata*, and ii) non-normalized and Illumina paired-end sequenced cDNA pools, four from roots and one from leaves (set #2B) of *Q. robur* clone DF159 [22],
- Sets #3, #4 and #5 consisted of newly sequenced Sanger, Roche 454 and Illumina reads as follows.

#### Targeted sequencing of putative “full-length” cDNA clones

Set #3 consisted of reads enriched in full-length (FL) cDNAs. Only trimmed Sanger ESTs from Ueno *et al*. [8] were included in this third set. For EST clones containing reads in both directions (5’ and 3’), overlapping contigs were assembled with CAP3 [69]. When the 5’ and 3’ ends of the same EST clone were sequenced and no overlap occurred, pseudocontigs were constructed by filling in the missing region of the EST clone with a 20 bp stretch of Ns. Singlets were defined as ESTs with only one read from a single EST clone. Overall, 100,228 sequences (85,817 singlets, 11,179 contigs and 3,232 pseudocontigs) were aligned against the *Arabidopsis thaliana* (*At*) protein sequences available from uniprotKB (http://www.uniprot.org/taxonomy/3702), with Blastx (e-value cutoff 1e^−10^). The high-scoring segment pair (HSP) of the top Blast hit was identified as the FL cDNA candidate when the alignment with an HSP started at the first methionine of the *At* protein. This analysis resulted in the detection of 6,910 FL cDNA candidates, 6,571 of which satisfied the conditions for expected insert size. In a second step, an equimolar pool of the 6,571 oak FL PCR fragments, at a final concentration of 10^7^ copies/μl, was prepared by pooling PCR amplicons from standard PCR carried out with M13 forward and reverse primers. The mean and median fragment sizes of the PCR products (as estimated on agarose gel) were 1,382 bp and 1,212 bp, respectively. DNA concentrations initially ranged from 80 to 150 ng/μl and were adjusted to 80 ng/μl, and aliquots of 3 μl of each probe were pooled in the same vial, with a Tecan Genesis RSP 200 liquid handling workstation (Tecan, Triangle Park, NC, USA), resulting in a total volume of 19.7 ml. This pool was split into 1 ml aliquots and DNA was precipitated by adding 3 M sodium acetate, pH 5.2 and 0.7 volumes of isopropanol. The DNA was collected by centrifugation, the pellet was dried and resuspended in 50 μl MilliQ water and the DNA was cleaned with a QIAquick PCR purification kit (Qiagen, Valencia, USA, CA). DNA concentration was measured with a Nanodrop 2000 spectrophotometer (Nanodrop Technologies, Wilmington, DE, USA). Finally, 75 bp paired-end sequencing was performed with a Genome Analyzer II x-sequencer (Illumina, San Diego, CA, USA), according to the manufacturer’s specifications.

#### Extension of the EST catalog with tissues challenged with abiotic and biotic stresses

Set #4 aimed to expand the diversity of expressed genes by sampling tissues subjected to abiotic and biotic challenges that had not been considered in previous studies. Two *Q. petraea* cDNA libraries were constructed from the pooled leaves or roots of six-month-old seedlings exposed to five abiotic stressors (10°C for 3 days, 35°C for 4 days, 700 ppm CO_2_, water stress, and hypoxia for 48 h). Six *Q. robur* libraries were established as described in Additional file 1, from a pool of control and treated seedlings subjected to biotic stressors, such as insect herbivory (gypsy moth *Lymantria dispar*), a fungal pathogen (powdery mildew *Erysiphe alphitoides*) and an oomycete pathogen (*Phytophthora cinnamomi*).

#### High-throughput sequencing from a tissue panel

We used the Illumina Hiseq2000 platform to sample genes with low levels of expression. Six tissues were studied: vegetative buds at two developmental stages (ecodormancy and swelling bud before bud break), differentiating secondary xylem, root, leaf, and dedifferentiated *in vitro* callus tissues (referred to as set #5). Total RNA was extracted as previously described [70]. We isolated mRNA by selection for the polyA tail. It was then chemically fragmented and converted into single-stranded cDNA by random hexamer priming. The second strand was then generated to create double-stranded cDNAs. Paired-end libraries were prepared according to the Illumina protocol (TruSeq Illumina DNA sample prep kit, Illumina, San Diego, CA, USA). Briefly, fragments were end-repaired, 3’-adenylated, and ligated to Illumina adapters. DNA fragments (with adapters) of 300–600 bp were amplified by PCR with Illumina adapter-specific primers. Libraries were quantified with a Qubit Fluorometer (Invitrogen, Milan, Italy). Library profiles were evaluated with an Agilent 2100 bioanalyzer (Agilent Technologies, Inc., Santa Clara, CA, USA). Each library was sequenced by 101 base-read length chemistry, in a paired-end flow cell, on the Illumina HiSeq2000 (Illumina, San Diego, CA, USA). Three libraries per lane were pooled to obtain about 130 million sequences per tissue type.

### Sequence processing

#### Sequences from the putative “full-length” cDNA-enriched library (set #3)

After base-calling with Phred [71], we eliminated vector and adapter sequences with cross_match (http://www.phrap.org/phredphrap/general.html), with the following parameters: −minmatch 10 -minscore 15. The vector database contained five vector sequences: pBluescriptSK (−) (X52324.1), a phagemid excised from lambda ZAP, pCR4-TOPO (Invitrogen, Carlsbad, CA, USA), pDNR-LIB, PDONR222.T, pGM-T_Easy. Low-complexity regions (mononucleotide repeats, PolyA) were then masked with RepeatMasker [72]. Using cross_match (−minmatch 10 -minscore 25), we then eliminated contaminants by comparison with several sequence databases, including Univec, and databases for the budding yeast and *E. coli* genomes. Valid sequences, with a PHRED-score of more than 20 over at least 100 bp lengths (to exclude potentially uninformative sequences) were retained for further analysis.

#### Sanger and 454 sequences (sets #1, #2A and #4)

All available Sanger ESTs were retrieved from the SURF database (http://genotoul-contigbrowser.toulouse.inra.fr:9092/Quercus_robur) and trimmed with Seqtrim 0.110 [73] to remove the library-specific cloning vector, to mask low-complexity sequences and to eliminate contaminants, mitochondrial sequences and poor-quality sequences. All 454 Roche sequences from this and previous [8,9] studies were cleaned up with SeqtrimNext 2.0.59 (http://www.scbi.uma.es/ingebiol/session/new/seqtrimnext).

#### Illumina sequences (sets #2B and #5)

Illumina paired-end reads were cleaned in a three-step procedure: i) sequencing adapters and low-quality nucleotides (quality value < 20) were removed, ii) sequences between the second unknown nucleotide (N) and the end of the read were removed, iii) reads shorter than 30 nucleotides after trimming were discarded, together with reads and their mates mapping onto run quality control sequences (PhiX genome).

### *De novo* transcript assembly

A schematic diagram of data processing for this study is shown in Figure 1. Short reads and long reads were subjected to different bioinformatic treatments, as described below.

#### Assembly of Illumina reads from putative full-length cDNAs (set #3)

A total of 17,196,106 Illumina paired-end reads bearing no similarity to vector sequences were assembled with Velvet V1.1.03 [74] and kmers 37, 41, 45, 49, 53, 57 and 61. A meta-assembly of the resulting contigs of more than 100 bp in length was then generated with TGICL V2.1 [75]. In total, 4,359 contigs were generated from the 6,571 cDNA clones initially amplified, and these contigs were subsequently cleaned up, with the removal of the remaining vector sequences with crossmatch (−minmatch 10 -minscore 15).

#### Pre-assembly of long reads (sets #1, #2A and #4)

We used MIRA V3.4.0 [76,77] to assemble 75,957 Sanger sequences, 2,790,004 Roche 454 reads and the *de novo* Illumina pre-assembly of 4,359 contigs. Contigs of less than 100 bp in length were filtered out. The CD-hit-EST V4.5.4 clustering algorithm [78,79] was used to reduce redundancy within this long-read pre-assembly (sequence identity threshold 0.95; word length 8). BLAT V34 was then used to validate this assembly, by mapping the initial Sanger and Roche-454 reads onto the long-read contigs. The minimum identity threshold was set to 98%.

#### Pre-assembly of short reads (sets #2B and #5)

The *de novo* assembly of the whole dataset was time-consuming due to memory issues, so we used Diginorm (Digital normalization with khmer, [80]) to normalize the raw data digitally. This process greatly decreases the size of shotgun data sets and the memory and time requirements for *de novo* sequence assembly, with no significant impact on the contigs generated. Diginorm was used to eliminate redundant reads. The coverage of each read was estimated (kmer-based approach) and reads with a coverage of less than 20 x were retained. Reads were assembled with Velvet V1.2.07 and Oases V0.2.8, using kmer 51. Potential fungal sequence contamination was identified by aligning the contigs with the sequences in the NCBI GenBank non-redundant protein sequence database (release 21/11/2012) with Blastx V2.2.15 (e-value cutoff 1e^−04^). Redundancy was reduced with CD-hit-EST (sequence identify threshold 0.95; word length 8).

#### Short- and long-read meta-assembly

The contigs from the long- and short-read pre-assemblies were assembled with MIRA V3.4.0, resulting in a final assembly named oak contigs v3 (OCV3) to distinguish it from two previous assemblies (OCV1 from [8], OCV2 from [22]). Redundancy among contigs was decreased with CD-hit-EST (sequence identity threshold 0.95; word length 8). We then filtered out contigs of less than 100 bp in length. We estimated chloroplast and mitochondrial contamination by BLAST searches with blastall V2.2.26 (e-value cutoff 1e^−5^) against the chloroplast genome of oak and a set of 162 contigs considered to correspond to the mitochondrial genome (both kindly provided by GG Vendramin, Institute of Biosciences and Bioresources, CNR, Sesto Fiorentino, Florence, Italy).

### Functional annotation and categorization of the oak proteome by comparative genomics

We compared the 192,097 oak contigs (referred to as OCV3-192 k) with six protein datasets, including Swissprot (release 02–2013) [81] and five plant proteomes: *Prunus persica* V1.0 (27,864 proteins, [82]), *Populus trichocarpa* V2.0 (40,668 proteins, [83]), *Vitis vinifera* V1.0 (26,346 proteins, [84]), *Eucalyptus grandis* V1.1 (36,376 proteins, [85]), *Arabidopsis thaliana* V9.0 (27,416 proteins, [86]). We used the BlastX program implemented in the blast + tool [87]. For each database, alignments with a score greater than 300 (BLOSUM62, gapo = 10, gape = 1, e-value = 1e10^−5^) were retained and the best alignment was used to identify the probable open reading frame to be considered for subsequent analysis. On the basis of these criteria, 90,786 oak transcripts (OCV3-91 k) were retained.

We established a first set of Gene Ontology (GO) terms [88] based on the best hit with Swissprot and the *At* proteome. We retrieved the GO terms associated with Swissprot and TAIR best hits from the Gene Ontology Annotation (GOA) project [89]. A second set of GO terms was associated with OCV3-91 K contigs by comparison of the 90,786 sequences with the Pfam V27.0 protein family database, using InterProScan V4.8 [90,91]. The GO terms were mapped onto plant GOslim terms with Blast2GO software [92]. The ontology level was set to 2. Due to the computational limitations of Blast2GO software, we retained only GO terms associated with at least 100 contigs.

### Identification of orthologous gene pairs between *Quercus* and malvids/fabids: inference of the timing of speciation

OCV3-91 k was aligned (BlastX, best match, e-value = 1e^−10^) with gene models for *Prunus persica*, *Populus trichocarpa*, *Vitis vinifera*, *Eucalyptus grandis* and *Arabidopsis thaliana*. The calculation of K*s* values (rate of synonymous substitution) between these contigs and gene models required sequences without stop codons and degenerate bases. We therefore translated the oak contigs into their six open reading frames and those without stop codons and degenerate bases were retained. The ClustalW [93] and PAML [94] packages were then used to calculate K*s*.

### Detection of genes differentially expressed in different tissues and at different stages of vegetative bud dormancy release, enrichment analysis

The BWA V 0.6.1 aligner was used to map Illumina paired-end reads onto the OCV3 assembly. If one or both paired reads mapped to the same contig, the result was recorded as a hit. When two reads from the same pair mapped to different contigs, they were not considered to constitute a hit. For the identification of contigs differentially expressed between the six Illumina libraries (ecodB: ecodormant buds, swB: swelling buds, XY: differentiating secondary xylem, RO: roots, LE: leaves, CA: *in vitro* dedifferentiated callus of *Q. robur* DF159 clone [95]), we assumed that the number of reads mapping onto a contig was proportional to the level of expression of the corresponding gene.

We first clustered the six tissues on the basis of their transcriptomic distances, by Ward’s linkage method [96]. The two pairwise distance metrics used (Pearson’s correlation coefficient and Euclidean distance) gave essentially identical results, so the results for only one of these methods are presented in the results section. Hierarchical clustering was performed with the R package *pvclust* [97] and the robustness of the clusters was assessed by multiscale bootstrap resampling (10,000) to obtain unbiased *p*-values. We used Log_2_(n + 1) normalized RPKM data (reads per kilobase of exon model per million mapped reads) to take contigs with no mapped read into account.

We applied different statistical tests to raw count, to detect differentially expressed genes: i) R statistics [98], ii) DESeq [99] and iii) EdgeR [100]. The last two of these methods were performed with the Bioconductor package. In R statistics, each contig was associated with an R value and was considered to be significantly differentially expressed with a type-I error risk of 2% if R ≥ 8. As there were no replicated libraries from which to estimate biological variability, we set the dispersion to 0.6 in the EdgeR package and used the exact test method. We applied a false discovery rate (FDR) of 0.05 with both these software suites. Contigs identified as significantly differentially expressed with at least two of the three methods were retained for further biological interpretation. Given the high rate of validation (based on RT-qPCR) of the *in silico* expression data obtained in a previous study based on the RNA-seq approach with stringent statistical criteria [32], we considered the data generated in this study to be accurate for the prediction of gene expression *in vitro*.

We then carried out an enrichment analysis for pathways and groups (EAPG) for selected genes differentially expressed between the ecodB and swB libraries, corresponding to two different phenological phases of vegetative bud release, with Pathway Studio 9 Desktop edition Software and the Resnet Plant Version 4 database (www.elsevier.com/online-tools/pathway-studio/plant-database). EAPG executes a Fisher’s exact test on each pathway or group and returns information relating to overlapping entities, together with the *p*-value of the statistical test. EAPG was executed against Gene Ontology and AraCyc plant metabolic pathway [101,102] data. The “Find subnetworks enriched with selected entities” function (FSNE) was also used to identify the set of entities (subnetworks) organized by specific relationships, with Fisher’s exact test.

### WEB portal

*Quercus* portal: https://w3.pierroton.inra.fr/QuercusPortal/.

## Additional files

Additional file 1:
**Oak** **(** ***Quercus petraea*** **and** ***Q. robur*** **)** **cDNA libraries used for Sanger, 454 Roche and Illumina sequencing.**
Additional file 2:
**Detailed procedure of short-reads, long-reads and meta-assemblies.**
Additional file 3:
**Distribution of trimmed cDNA length (Sanger (blue) and Roche 454 (orange) sequences) used in the long-read assembly.** *y*-axis: number of ESTs within different categories of trimmed sequence length. *x*-axis: ranges of trimmed sequence lengths (101–200, 201–300, 301–400 bp, etc.). Additional file 4:
**Cumulative contig size for the short- (red), long- (black) and meta- (green) assemblies.** *y*-axis: Number of contigs (%). *x*-axis: contig sizes on a log_10_ scale. Additional file 5:
**List of GO terms associated with OCV3-91 k contigs.**
Additional file 6:
**Number of OCV3 contigs associated with the main GO terms (BP: Biological Process, CC Cellular Component, MF Molecular Function) and distribution of GO terms at the second level of the Gene Ontology.**
Additional file 7:
**Comparison of GO term rankings between this study and three other unigene sets, for** ***Castanea dentata,*** ***Eucalyptus grandis,*** ***Pseudotsuga menziesii.***
Additional file 8:
**List of 9,549 oak contigs with single robust orthologs in the** ***Prunus persica*** **gene model.**
Additional file 9:
**Information gained by sequencing several tissues.** Abbreviation: RO: root, ecodB: ecodormant bud, LE: leaf, CA: *in vitro* dedifferentiated callus, swB: swelling bud, XY: secondary differentiation xylem. In the OCV3-91 k subset, 2,275 contigs (2.5%) contained no reads from the tissue panel. Additional file 10:
**Number of paired-reads, reads mapping to OCV3-91 k, mapped contigs and “tissue-specific” contigs. Abbreviation: ecodB: ecodormant bud, swB: swelling bud, XY: secondary differentiation xylem, RO: root, LE: leaf and CA:** ***in vitro*** **dedifferentiated callus.**
Additional file 11:
**List of “tissue-specific” contigs in the OCV3-91 k subset.**
Additional file 12:
**Normalized read counts for OCV3-91 K and OCV3-101 K as a result of the mapping of the 6 illumina libraries (RO, ecoDB, LE, CA, swB and XY).**
Additional file 13:
**Dendrogram of the distances between six tissues (ecodB: ecodormant bud, swB: swelling bud, XY: differentiating secondary xylem, RO: root, LE: leaf and CA:** ***in vitro*** **dedifferentiated callus), constructed by Ward’s linkage method with Euclidean distance as the dissimilarity metric.** The axis next to the tree indicates the mean distance (inverse of similarity) between members of the two branches joined at each node. The robustness of the clusters was assessed by multiscale bootstrap resampling (10,000) to obtain unbiased *p*-values. In red: AU *p*-values (approximately unbiased, Multiscale bootstrap resampling), in green: BP values (bootstrap probability). Additional file 14:
**List and annotation of 23 OCV3 contigs differentially expressed, as shown by three statistical methods (R statistics, EdgeR and DESeq), between ecodB (ecodormancy) and swB (swelling bud) libraries.**
Additional file 15:
**List and annotation of 862 differentially expressed OCV3 contigs (663 contigs from OCV3-91 k and 199 contigs from OCV3-101 k) identified by at least two statistical methods (R statistics, EdgeR, and DESeq).**
Additional file 16:
**Result of the enrichment analysis for pathways and groups.**
