# Supplementary material for: The oak gene expression atlas: insights into Fagaceae genome evolution and the discovery of genes regulated during bud dormancy release
Source: BMC Genomics. 2015 Feb 21;16(1):112. doi: 10.1186/s12864-015-1331-9 (PMC4350297; doi:10.1186/s12864-015-1331-9)
Supplement: Additional file 2: — Detailed procedure of short-reads, long-reads and meta-assemblies. [file 12864_2015_1331_MOESM2_ESM.pdf]

Additional file 2: Detailed procedure of short-reads, long-reads and meta-assemblies.

*Long-read pre-assembly*

The sequencing of 29 and 36 cDNA libraries with Sanger and 454 technologies (sets #1, #2A and #4 in Figure 1) resulted in 94,174 and 2,790,004 reads with a mean length of 526 and 336 bp, respectively, after the removal of vector sequence, low-quality sequences and duplicated reads. The distribution of trimmed ESTs is shown in Additional file 3. The 6,571 putative FL-cDNA clones (set #3) were sequenced on an Illumina/Solexa GA-II X, yielding 32,726,953 initial read pairs (75 bp), 25,140,399 of which presented little or no overlap with the vector sequence and were retained for further analysis. In total, 17,196,106 pairs (68.4%) were aligned with the corresponding putative FL-cDNAs. At least one pair of the reads aligned with 6,533 initial ESTs, and at least 10 pairs aligned with 6,458 initial ESTs, indicating that 98.3% of the PCR-amplified clones gave reasonable alignments with Illumina reads. The *de novo* assembly of these Illumina paired-end reads with Velvet and TGICL software yielded 4,359 contigs, 47% of which were longer and 53% of which were shorter than the initial EST. This suggests that Illumina reads were not evenly distributed within cDNA clones.

By combining Sanger, Roche-454 and reconstructed FL-cDNA data, we obtained 2,888,537 long sequences, 2,003,295 of which were used to construct a long-read pre-assembly with MIRA. We obtained 69,982 contigs and 300,373 singletons ($\geq$ 100 bp). The use of CD-HIT-EST to decrease sequence redundancy in the set of contigs resulted in a final set of 44,279 contigs. A Blast-like alignment tool (BLAT) was used to validate this pre-assembly, by mapping Sanger, Roche-454 and FL cDNA reads onto the assembly. This final step was carried out with a minimum identity threshold of 98%, and led to the validation of 44,272 contigs, with a mean sequence length of 937 bp (standard deviation: 521 bp; N50: 1,118 bp, defined as the largest entity E such that at least half of the total size of the entities is contained in entities larger than E; see the black curve in Additional file 4).

*Short-read pre-assembly*

In total, 961,151,725 Illumina reads (set #2B and #5) were initially available. Digital normalization with Diginorm decreased the size of this dataset to 78,300,315 reads (8.15% of the initial dataset), 38,181,170 of which were assembled into 352,384 contigs with Velvet and Oases. We then removed contigs displaying significant similarity to fungal sequences and decreased redundancy with CD-hit-EST, to generate a short-read pre-assembly of 230,595 contigs with a mean sequence length of 877 bp (SD 1,069 bp; N50 1,758bp, red curve in Additional file 4).

*Meta-assembly*

The meta-assembly was generated with MIRA. Of the 274,867 pre-assembly contigs (44,272 long-read contigs and 230,595 short-read contigs), 128,214 pre-assembled contigs were included in 48,672 newly extended contigs. Of the non-extended contigs, 12,898 and 133,755 belonged to the initial long- and short-read pre-assemblies, respectively. After redundancy reduction with CD-hit-EST, these 195,325 contigs merged into 192,098 contigs, 48,208 of which corresponded to newly constructed contigs, whereas10,899 and 132,990 belonged to the initial long- and short-read pre-assemblies, respectively. Finally, after filtering out contigs shorter than 100 bp, we obtained a final assembly (OCV3) consisting of 192,097 contigs, which is available from the Quercus portal (https://w3.pierroton.inra.fr/QuercusPortal/index.php?p=est_OCV3_TEMP). In total, 1,623 (0.84%) and 2,747 (1.43%) contigs yielded significant hits with the oak chloroplast and mitochondrial genomes, respectively. The mean contig size for OCV3 was 1,037 bp (SD 1,150 bp; N50 1,879 bp, green curve in Additional file 4), which is close to the mean gene length in eukaryotes (1,346 bp, [22]). By assembling short and long reads together in a single unigene set, we were able to improve the first oak transcriptome assembly (OCV1) established by Ueno *et al.* 2010 [8] from Sanger and Roche-454 reads. Indeed, a systematic comparison between OCV1 and OCV3 (Table 1B) showed that N50 increased from 908 to 1,879 bp and that the number of uniquely identified SwissProt IDs increased from 13,333 to 17,476. On the other hand, a comparison of OCV3 with the OCV2

assembly of 65,712 contigs from the *Q. robur* genotype DF159 (Table 1B, [23]) showed an increase in N50 from 1,545 bp to 1,879 bp and an increase in the number of unique SwissProt IDs from 16,429 to 17,476. It is difficult to compare the size of the meta-assembly (about 192 thousand contigs) with those of other projects with similar aims and approaches, because it is influenced by genome and transcriptome sizes, the diversity of tissues/developmental stages/environmental conditions, the number of cDNA sequences produced, and the assembly method used. However, if we consider recent studies on forest trees, the OCV3 meta-assembly is of a similar size to those of *Pseudotsuga menziesii* (170,859 contigs [24]) and *Pinus pinaster* (210,513 contigs [25]), larger than that of *Castanea molissima* (40,039 contigs [26]), and smaller than of *Pinus contorta* (303,450 contigs, [27]).
