# Supplementary figures and images for: The oak gene expression atlas: insights into Fagaceae genome evolution and the discovery of genes regulated during bud dormancy release
Source: BMC Genomics. 2015 Feb 21;16(1):112. doi: 10.1186/s12864-015-1331-9 (PMC4350297; doi:10.1186/s12864-015-1331-9)

Number of sequences

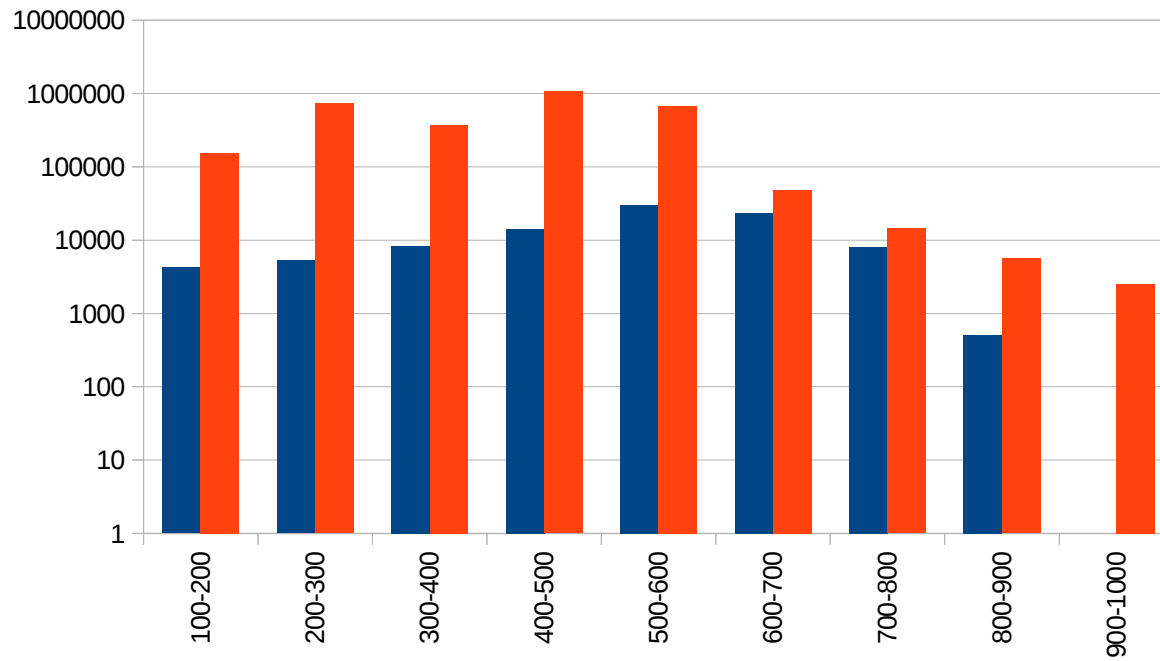

Trimmed sequence lengths

Supplement: Additional file 3: — Distribution of trimmed cDNA length (Sanger (blue) and Roche 454 (orange) sequences) used in the long-read assembly. y-axis: number of ESTs within different categories of trimmed sequence length. x-axis: ranges of trimmed sequence lengths (101–200, 201–300, 301–400 bp, etc.). [file 12864_2015_1331_MOESM3_ESM.pdf]
