# Supplementary material for: The oak gene expression atlas: insights into Fagaceae genome evolution and the discovery of genes regulated during bud dormancy release
Source: BMC Genomics. 2015 Feb 21;16(1):112. doi: 10.1186/s12864-015-1331-9 (PMC4350297; doi:10.1186/s12864-015-1331-9)
Supplement: Additional file 4: — Cumulative contig size for the short- (red), long- (black) and meta- (green) assemblies. y-axis: Number of contigs (%). x-axis: contig sizes on a log10 scale. [file 12864_2015_1331_MOESM4_ESM.ppt]

## Slide 1
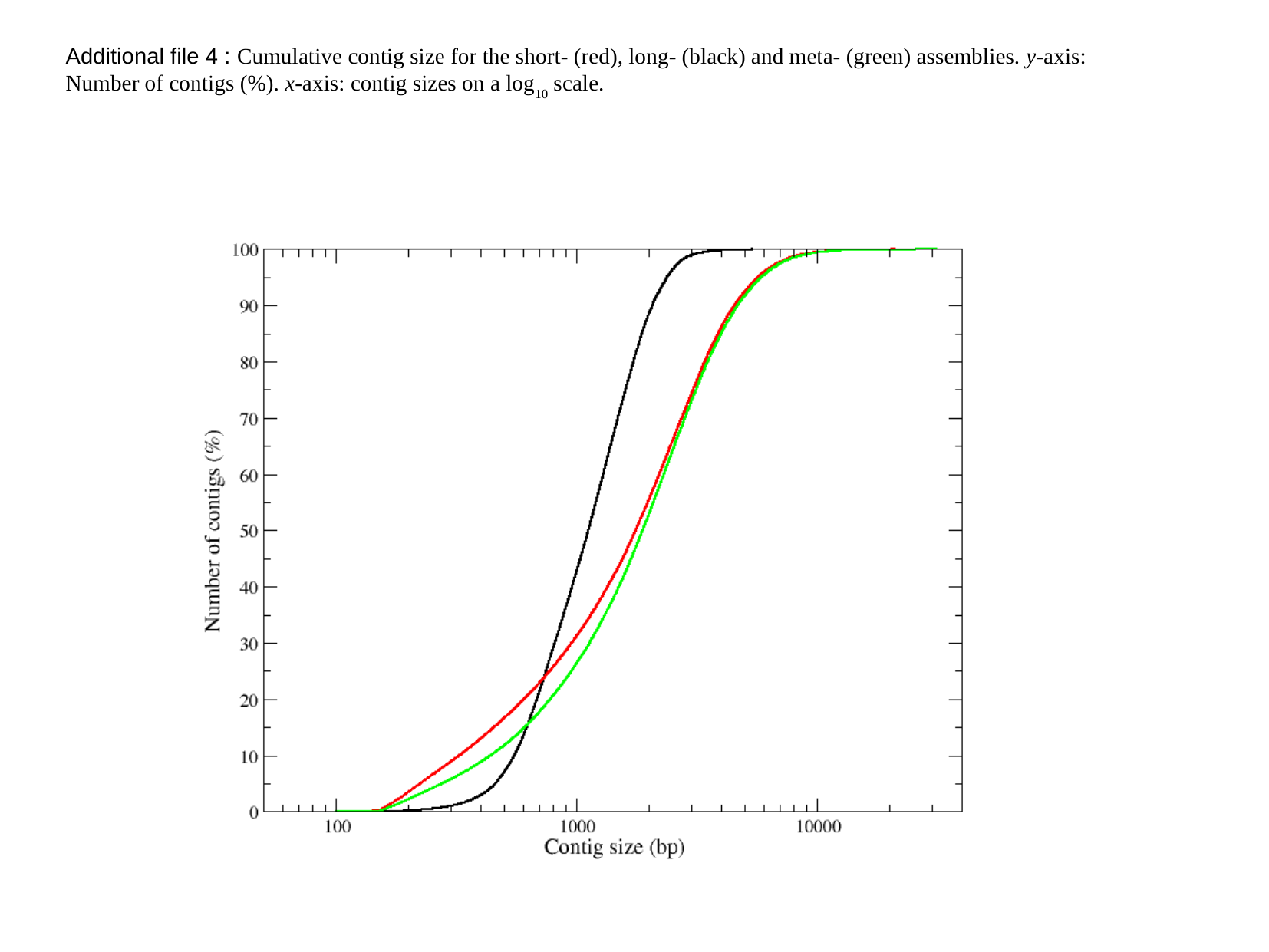

Additional file 4 : Cumulative contig size for the short- (red), long- (black) and meta- (green) assemblies. y-axis: Number of contigs (%). x-axis: contig sizes on a log10 scale.
