# Supplementary material for: The oak gene expression atlas: insights into Fagaceae genome evolution and the discovery of genes regulated during bud dormancy release
Source: BMC Genomics. 2015 Feb 21;16(1):112. doi: 10.1186/s12864-015-1331-9 (PMC4350297; doi:10.1186/s12864-015-1331-9)

Additional file 9: Information gained by sequencing several tissues. Abbreviation: RO: root, ecodB: ecodormant bud, LE: leaf, CA: *in vitro* dedifferentiated callus, swB: swelling bud, XY: secondary differentiation xylem. In the OCV3-91k subset, 2,275 contigs (2.5%) contained no reads from the tissue panel.

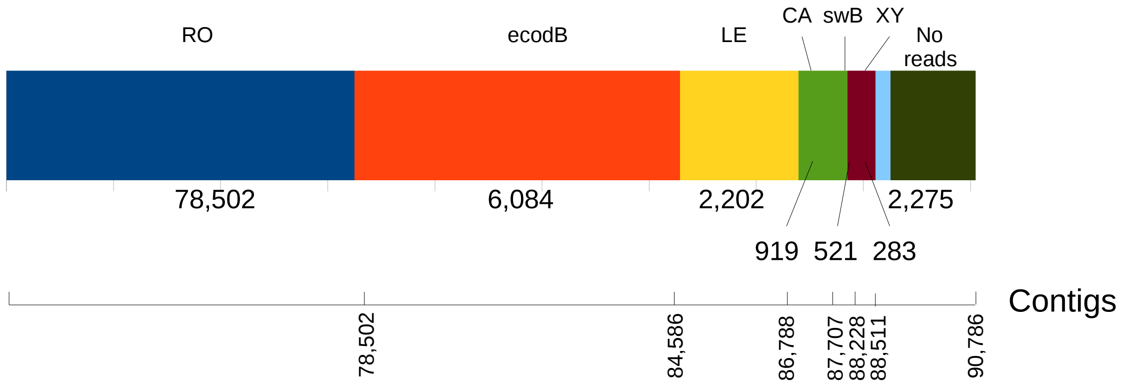

Supplement: Additional file 9: — Information gained by sequencing several tissues. Abbreviation: RO: root, ecodB: ecodormant bud, LE: leaf, CA: in vitro dedifferentiated callus, swB: swelling bud, XY: secondary differentiation xylem. In the OCV3-91 k subset, 2,275 contigs (2.5%) contained no reads from the tissue panel. [file 12864_2015_1331_MOESM9_ESM.pdf]
