# Supplementary material for: The oak gene expression atlas: insights into Fagaceae genome evolution and the discovery of genes regulated during bud dormancy release
Source: BMC Genomics. 2015 Feb 21;16(1):112. doi: 10.1186/s12864-015-1331-9 (PMC4350297; doi:10.1186/s12864-015-1331-9)
Supplement: Additional file 10: — Number of paired-reads, reads mapping to OCV3-91 k, mapped contigs and “tissue-specific” contigs. Abbreviation: ecodB: ecodormant bud, swB: swelling bud, XY: secondary differentiation xylem, RO: root, LE: leaf and CA: in vitro dedifferentiated callus. [file 12864_2015_1331_MOESM10_ESM.doc]

**Additional file 10** Number of paired-reads, reads mapping to OCV3-91k, mapped contigs and tissue-specific contigs. Abbreviation: ecoDB: ecodormant bud, swB: swelling bud, XY: secondary differentiating xylem, RO: root, LE: leaf and CA: *in vitro* dedifferentiated callus.

| Library | Nb of paired- reads | Nb of successfully mapped reads on OCV3-91K | Nb of OCV3-91K contigs | Nb of specific OCV3-91K contigs |
| --- | --- | --- | --- | --- |
| **ecodB** | 59,050,722 | 19,401,894 | 74,493 (82.05%) | 1,039 (1.14%) |
| **swB** | 63,191,029 | 21,960,649 | 74,254 (81.79%) | 341 (0.38%) |
| **XY** | 68,158,203 | 22,344,400 | 73,240 (80.67%) | 283 (0.31%) |
| **RO** | 72,263,408 | 22,705,763 | 78,502 (86.47%) | 1,756 (1.93%) |
| **LE** | 57,005,112 | 16,921,980 | 77,001 (84.82%) | 977 (1.08%) |
| **CA** | 65,878,896 | 19,141,440 | 69,964 (77.06%) | 514 (0.57%) |
