# Supplementary material for: The oak gene expression atlas: insights into Fagaceae genome evolution and the discovery of genes regulated during bud dormancy release
Source: BMC Genomics. 2015 Feb 21;16(1):112. doi: 10.1186/s12864-015-1331-9 (PMC4350297; doi:10.1186/s12864-015-1331-9)
Supplement: Additional file 13: — Dendrogram of the distances between six tissues (ecodB: ecodormant bud, swB: swelling bud, XY: differentiating secondary xylem, RO: root, LE: leaf and CA: in vitro dedifferentiated callus), constructed by Ward’s linkage method with Euclidean distance as the dissimilarity metric. The axis next to the tree indicates the mean distance (inverse of similarity) between members of the two branches joined at each node. The robustness of the clusters was assessed by multiscale bootstrap resampling (10,000) to obtain unbiased p-values. In red: AU p-values (approximately unbiased, Multiscale bootstrap resampling), in green: BP values (bootstrap probability). [file 12864_2015_1331_MOESM13_ESM.ppt]

## Slide 1
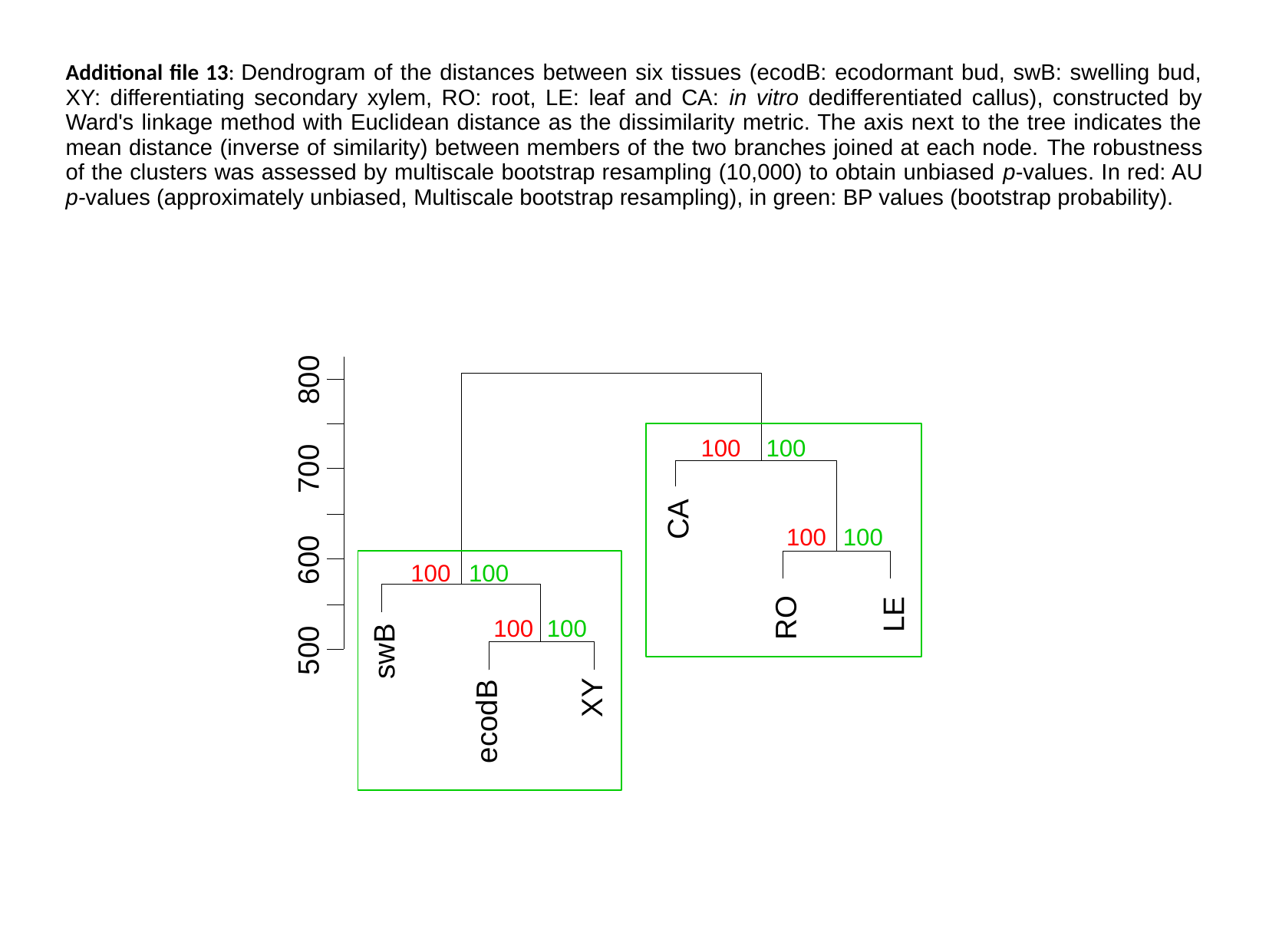

Additional file 13: Dendrogram of the distances between six tissues (ecodB: ecodormant bud, swB: swelling bud, XY: differentiating secondary xylem, RO: root, LE: leaf and CA: in vitro dedifferentiated callus), constructed by Ward's linkage method with Euclidean distance as the dissimilarity metric. The axis next to the tree indicates the mean distance (inverse of similarity) between members of the two branches joined at each node. The robustness of the clusters was assessed by multiscale bootstrap resampling (10,000) to obtain unbiased p-values. In red: AU p-values (approximately unbiased, Multiscale bootstrap resampling), in green: BP values (bootstrap probability).
